# Quantification of protein cargo loading into engineered extracellular vesicles at single‐vesicle and single‐molecule resolution

**DOI:** 10.1002/jev2.12130

**Published:** 2021-08-02

**Authors:** Andreia M. Silva, Elisa Lázaro‐Ibáñez, Anders Gunnarsson, Aditya Dhande, George Daaboul, Ben Peacock, Xabier Osteikoetxea, Nikki Salmond, Kristina Pagh Friis, Olga Shatnyeva, Niek Dekker

**Affiliations:** ^1^ Discovery Biology Discovery Sciences BioPharmaceuticals R&D AstraZeneca Gothenburg Sweden; ^2^ Advanced Drug Delivery Pharmaceutical Sciences BioPharmaceuticals R&D AstraZeneca Gothenburg Sweden; ^3^ Structure and Biophysics Discovery Sciences BioPharmaceuticals R&D AstraZeneca Gothenburg Sweden; ^4^ NanoView Biosciences Boston Massachusetts USA; ^5^ NanoFCM INC. Nottingham UK; ^6^ Discovery Biology Discovery Sciences BioPharmaceuticals R&D AstraZeneca Alderley Park UK

**Keywords:** EV cargo sorting, exosomes, ExoView, extracellular vesicles, nanoflow cytometry, protein delivery vehicle, single‐molecule localization microscopy

## Abstract

Extracellular Vesicles (EVs) have been intensively explored for therapeutic delivery of proteins. However, methods to quantify cargo proteins loaded into engineered EVs are lacking. Here, we describe a workflow for EV analysis at the single‐vesicle and single‐molecule level to accurately quantify the efficiency of different EV‐sorting proteins in promoting cargo loading into EVs. Expi293F cells were engineered to express EV‐sorting proteins fused to green fluorescent protein (GFP). High levels of GFP loading into secreted EVs was confirmed by Western blotting for specific EV‐sorting domains, but quantitative single‐vesicle analysis by Nanoflow cytometry detected GFP in less than half of the particles analysed, reflecting EV heterogeneity. Anti‐tetraspanin EV immunostaining in ExoView confirmed a heterogeneous GFP distribution in distinct subpopulations of CD63^+^, CD81^+^, or CD9^+^ EVs. Loading of GFP into individual vesicles was quantified by Single‐Molecule Localization Microscopy. The combined results demonstrated TSPAN14, CD63 and CD63/CD81 fused to the PDGFRβ transmembrane domain as the most efficient EV‐sorting proteins, accumulating on average 50–170 single GFP molecules *per* vesicle. In conclusion, we validated a set of complementary techniques suitable for high‐resolution analysis of EV preparations that reliably capture their heterogeneity, and propose highly efficient EV‐sorting proteins to be used in EV engineering applications.

## 1 INTRODUCTION

Extracellular vesicles (EVs) are nanosized particles secreted by virtually all cell types to the extracellular space or in body fluids, conveying inter‐cellular communication by functional transfer of biomolecules (Kalluri & LeBleu, 2020). EVs have been explored as natural delivery vehicles of therapeutic drug molecules used to treat human diseases (de Abreu et al., 2020; Negi et al., 2019; Shaimardanova et al., 2019; Walker et al., 2019). In fact, EVs have shown great potential as novel candidates for intracellular delivery of various types of therapeutic cargo, including synthetic small molecules and biological molecules such as RNA and large proteins (Luan et al., 2017). Furthermore, these vesicles have several advantages compared to synthetic delivery systems including small size, low immunogenicity, lack of cytotoxicity, long‐term safety, and amenability to cargo loading by a diversity of strategies (Elsharkasy et al., 2020; Saleh et al., 2019).

EVs can be engineered to modify their cargo composition with molecules of interest (Armstrong et al., 2017). In particular, EV modification with a protein cargo is commonly achieved by cell engineering with plasmids that encode and overexpress the target protein fused to an intraluminal or membrane protein naturally incorporated in EVs during their biogenesis. The EV proteins selected for this purpose are usually enriched in EV preparations (Kowal et al., 2016), having the potential to promote selective cargo loading at high levels into the vesicles, and thus, were here designated as EV‐sorting proteins. Commonly used EV‐sorting proteins include tetraspanins (especially CD63, CD9 and CD81) (Heath et al., 2019; Li et al., 2019; Men et al., 2019), lamp2b (Alvarez‐Erviti et al., 2011) and the C1C2 domain of lactadherin (Delcayre et al., 2005). The fusion of the cargo protein of choice to EV‐sorting proteins is the most commonly reported strategy to load protein cargo into EVs via parental cell engineering. This approach allows for both intraluminal storage of cargo that can be delivered to target cells, as well as for cargo display at the EVs surface, to enhance their therapeutic signalling properties or targeting capacity, by direct interaction with target cell surface receptors. Protein cargoes reported to have been successfully loaded into EVs include luciferase and fluorescent proteins for EV tracking *in vitro* and *in vivo* (Betzer et al., 2020; Lázaro‐Ibáñez et al., 2021), antibodies and peptides for EV targeting (Longatti et al., 2018; Mentkowski & Lang, 2019), active enzymes (Ye et al., 2020), RNA‐binding proteins for simultaneous RNA loading into EVs (Hung & Leonard, 2016) and vaccine immunogens (Zeelenberg et al., 2008), amongst others. Interestingly, many of these studies showed that enrichment levels of cargo proteins loaded into EVs depend on the EV‐sorting protein used (Corso et al., 2019; Osteikoetxea et al., 2020). However, a comprehensive comparison of the loading efficiency of distinct EV‐sorting proteins is still limited to a few candidate proteins, being further impaired by a lack of tools for quantitative analysis of cargo loading.

There are multiple biogenesis routes in cells that result in the release of EVs, explaining the presence of heterogeneous populations of vesicles with distinct compositions in cell‐conditioned media (van Niel et al., 2018). Even the preparations of naïve or engineered EVs isolated by the most specific methodologies currently available are still heterogeneous and comprise subpopulations of vesicles with distinct composition (Kalluri & LeBleu, 2020). Evaluation of protein cargo loading into EVs is mostly limited to methods of bulk protein content analysis, such as Western blotting, Enzyme‐Linked Immunosorbent Assay (ELISA), conventional Flow cytometry of EVs coupled to beads, and Mass Spectrometry (Théry et al., 2018), all of which are not suitable for single‐vesicle characterisation. More recently, several techniques have been described for single‐vesicle analysis to allow for more accurate characterisation of protein cargo loading, with some of them reaching single‐molecule resolution. These comprise Transmission Electron Microscopy (TEM) coupled with immunogold labelling (Gärtner et al., 2019), Atomic Force Microscopy (Matsumura et al., 2019; Yuana et al., 2010), Super‐Resolution Fluorescence Microscopy (Nizamudeen et al., 2018), Fluorescence Correlation Spectroscopy (Corso et al., 2019), Nanoflow cytometry (Choi et al., 2019; Morales‐Kastresana et al., 2017), Imaging Flow cytometry (Görgens et al., 2019), Single‐Particle Interferometric Reflectance Imaging Sensing (SP‐IRIS) (Daaboul et al., 2016), and Laser tweezers Raman spectroscopy (Smith et al., 2015), amongst others (Gori et al., 2020; Lee et al., 2018). Importantly, these approaches have revealed a heterogeneous distribution of cargo across naïve and engineered EV subpopulations, highlighting the requirement of higher‐resolution techniques in the standard characterisation of EVs.

In this study, we aimed to explore and validate techniques for analysis of engineered EVs at the single‐vesicle and single‐molecule level, for a rapid and robust evaluation of the efficiency of different EV‐sorting proteins in promoting the loading of protein cargo. Expi293F cells were engineered to secrete EVs loaded with green fluorescent protein (GFP) fused to selected membrane‐associated proteins that are highly enriched in EVs. GFP levels in small EVs were analysed in bulk using the standard EV characterisation technique of Western blotting and compared to the novel high‐resolution single‐vesicle analysis methods Nanoflow cytometry, ExoView and Single‐Molecule Localization Microscopy (SMLM). Our findings validate Nanoflow cytometry using a NanoAnalyzer N30 device and ExoView as fast and reliable techniques for single‐vesicle analysis of engineered EVs, capturing the heterogeneous GFP distribution across EV subpopulations. SMLM was validated as the most accurate approach that allowed quantification of GFP copy number loaded in individual EVs. The comparative analysis of GFP enrichment levels upon fusion to different EV‐sorting proteins also revealed new candidates suitable for loading of protein cargo into EVs at high efficiency, expanding the range of EV‐sorting proteins that can be used for the generation of engineered EVs with enhanced capacity to target recipient cells, and to functionally deliver cargoes of therapeutic value.

## 2 METHODS

### 2.1 Selection and bioinformatic characterisation of EV‐sorting proteins

The databases Vesiclepedia (http://microvesicles.org) (Kalra et al., 2012), ExoCarta (http://exocarta.org) (Mathivanan & Simpson, 2009) and EVpedia (http://evpedia.info) (Kim et al., 2013) were queried for *Homo sapiens* EV protein content, and entries corresponding to proteins with less than 50 kDa for which commercial detection antibodies were available were further selected (Subset 1). A literature search was performed to select the proteins that tolerate fusions and that do not impact negatively EV biogenesis (Subset 2). EV protein and GFP sequences were obtained from UniProt (entry IDs: CD47: Q08722; SDCBP: O00560; APMAP: Q9HDC9; TSPAN14: Q8NG11; CD63: P08962; CD81: P60033; PDGFRβ: P09619; GFP: C5MKY7, carrying the mutations V2A, F65L, S66T and H232L for enhanced fluorescence) to generate fusion proteins (protein sequences available on Table S1). The membrane topology of the fusion proteins was predicted from UniProt annotations and literature previously published on EV‐sorting proteins.

### 2.2 Plasmid design

Protein sequences of EV‐sorting domains and GFP fusion proteins were reverse translated into a DNA sequence using codon optimization for protein expression in *Homo sapiens*. DNA inserts were synthesized and cloned into the pEBNAZ expression vector (modified from (Stewart et al., 2011)) at SacII and NotI restriction sites, for recombinant protein expression under the control of a CMV promoter. All constructs were sourced through GenScript.

### 2.3 Cell culture

Expi293F suspension cells, derived from 293F human embryonic kidney cells, were purchased from ThermoFisher Scientific. Cells were seeded at a density of 0.5–1 × 10^6^ cells/ml and grown to a density of 3–4 × 10^6^ cells/ml in chemically defined, serum‐free, protein‐free Expi293 expression media (ThermoFisher Scientific) at 37 °C, 8% CO_2_, at 150 rpm in 2 l Corning roller bottles (Sigma‐Aldrich). Cells were counted and viability was measured using a Cedex HiRes Analyzer (Roche Diagnostics). Cells were used until passage 21. All cells were mycoplasma negative and authenticated by short tandem repeat DNA profiling analysis.

### 2.4 Cell transfection for EV production

Expi293F cells were seeded at a density of 3.9 × 10^6^ cells/ml in 45 ml of Expi293 expression media in a 500 ml Corning Erlenmeyer cell culture flask. Plasmid DNA (75 μg) was diluted in Expi293 expression media and mixed in a 1:1 (v/v) ratio with 40 kDa linear polyethylenimine hydrochloride 120 μg/ml (Polysciences) in Expi293 expression media. This mix was incubated for 15 min at room temperature (RT), and then gently added to the cells. Cells treated in the same conditions with polyethylenimine complexes lacking DNA were used as untransfected control. After 24 h, 50 ml of fresh culture media was added to each cell culture flask. Cells were maintained at 37 °C, 8% CO_2_, 150 rpm.

### 2.5 Cell imaging and flow cytometry analysis

At 48 h post‐transfection, control and transfected Expi293F cells were fixed with paraformaldehyde 4% in phosphate buffered saline (PBS) (VWR) and nuclei were stained with Hoechst 33342 (ThermoFisher Scientific). Cells were then resuspended in PBS and transferred into a 96‐well CellCarrier Ultra plate (PerkinElmer), briefly spun down to sediment cells at the bottom of the wells, and immediately imaged in a spinning disk confocal CV7000 microscope (Yokogawa) using a 40x objective (NA 0.75), and lasers 405 nm (BP447/45 nm) and 488 nm (BP522/35 nm). Six fields were imaged *per* well, recording several stacks for each position. Images were processed using ImageJ 1.52p software. Fixed cells were also analysed by flow cytometry using a BD LSRFortessa flow cytometer equipped with BD FACSDiva software (BD Biosciences), acquiring 10,000 events in gated cells. Data was analysed using FlowJo v10.7.1 software (Becton, Dickinson & Company). GFP background fluorescence level of non‐transfected control cells was used as the threshold to define GFP^+^ cell populations.

### 2.6 EV isolation

After 48 h of cell transfection, cell culture supernatants were spun at 300 x *g* for 10 min to remove cells, and clarified supernatant was transferred to new tubes and further centrifuged at 2,500 x *g* for 30 min, 4 °C, to remove residual cell debris and apoptotic blebs. Next, the supernatant was transferred to polyallomer 94 ml Quick‐Seal ultracentrifuge tubes (Beckman Coulter) and centrifuged at 20,000 x *g* for 25 min, 4 °C, to remove large EVs. A 45Ti Rotor (Beckman Coulter) was used in an Optima XPN‐80 ultracentrifuge (Beckman Coulter). The supernatant was then transferred to new tubes and ultracentrifuged at 100,000 x *g* for 2 h, 4 °C, using the same rotor as above (K‐factor = 210.4). The resulting pellets were washed by resuspension in PBS, and re‐pelleted in the same conditions as above. EV pellets were resuspended in 100–200 μl PBS, aliquoted, and frozen at −80 °C until further use.

### 2.7 Transmission electron microscopy of EVs

EV suspensions were fixed for 30 min in paraformaldehyde 2% (Sigma‐Aldrich) in PBS. Carbon‐coated 100‐mesh copper grids were glow discharged at 7.2 V for 60 s, using a Bal‐Tec MED 020 Coating System, and EVs samples were immediately incubated on top of the grids for 15 min, at 4 °C. Grids were then washed in PBS, fixed in glutaraldehyde 1% (Sigma‐Aldrich) for 5 min at 4 °C, washed again and blotted dry with filter paper. EVs adsorbed onto the grids were negatively stained with aqueous uranyl acetate 2% (Sigma‐Aldrich) for 2 min, followed by washing, drying and analysis using a FEI Tecnai G2 Spirit transmission electron microscope (ThermoFisher Scientific), equipped with a Morada digital camera (Olympus Soft Image Solutions GmbH).

### 2.8 Nanoparticle tracking analysis of EVs

The size distribution and particle concentration of the EV preparations were analysed using NanoSight LM14c (Malvern Panalytical) equipped with a blue laser (488 nm, 70 mW) and a CMOS camera (Hamamatsu Photonics). Samples were diluted in PBS from thousand‐ to eight thousand‐fold for measurement of 20–100 particles/frame in all conditions, injected at a speed of 100 a.u. into the measuring chamber and EVs flow recorded in triplicate measurements of 90 s each, at 25 °C. Equipment settings for data acquisition were kept constant between measurements with camera level set to 15, auto settings off, screen gain set to one and threshold set to seven. Data analysis was performed with NTA 3.2 software (Malvern Panalytical).

### 2.9 Western blotting analysis of EVs and cell lysates

Cell pellets were collected at 48 h post‐transfection, washed with PBS and lysed with Pierce RIPA buffer (ThermoFisher Scientific) supplemented with cOmplete EDTA‐free protease inhibitor cocktail (Sigma‐Aldrich) for 30 min, on ice. Lysates were centrifuged at 14,000 rpm for 10 min at 4 °C, supernatant recovered and preserved at −20 °C. Protein concentration of EV suspensions and parental cell lysates was quantified using Qubit protein assay kit (ThermoFisher Scientific), according to the manufacturer's protocol. The same amount of total protein or particle number, as indicated, was then prepared for all samples in NuPAGE LDS sample buffer (ThermoFisher Scientific), for detection of CD63 and CD81 in non‐reducing conditions, with further supplementation with NuPAGE Reducing Agent for the detection of all other proteins in reducing conditions. Protein samples were denatured at 75 °C for 10 min and resolved on NuPAGE 4–12% Bis‐tris gels (ThermoFisher Scientific) in NuPAGE MES SDS running buffer (ThermoFisher Scientific). Proteins were transferred onto PVDF membranes (Bio‐Rad Laboratories), using a Trans‐Blot Turbo Transfer System (Bio‐Rad Laboratories). Membranes were blocked with Odyssey tris‐buffered saline (TBS) buffer (Li‐COR) for 1 h at RT, and incubated overnight at 4 °C with the following primary antibodies: chicken polyclonal anti‐GFP (ab13970, Abcam), mouse monoclonal anti‐CD63 (ab59479, Abcam), mouse monoclonal anti‐CD81 (ab79559, Abcam), rabbit polyclonal anti‐TSG101 (ab30871, Abcam), mouse polyclonal anti‐Alix (ab117600, Abcam), mouse monoclonal anti‐β‐actin (A1978, Sigma‐Aldrich) and rabbit polyclonal anti‐calnexin (ab22595, Abcam), all diluted 1:1,000 in Odyssey TBS buffer, except for anti‐β‐actin, diluted 1:2,000. Membranes were then washed three times with TBS‐Tween‐20 0.05% (TBS‐T; Sigma‐Aldrich) and incubated for 1 h at RT with anti‐mouse, anti‐rabbit or anti‐chicken fluorophore‐conjugated secondary antibodies (all from LI‐COR), diluted 1:20,000 in TBS‐T. Membranes were imaged on an Odyssey CLx imaging system (LI‐COR) equipped with Image Studio v4.0 software. Densitometry analysis was performed on ImageJ 1.52p software, for bands with a size corresponding to the predicted molecular weight of the fusion proteins.

### 2.10 EV labelling and analysis by Nanoflow cytometry

EVs (5 × 10^11^ particles/ml) were labelled with CellTrace Far Red dye 5 μM for 15 min, at RT. Excess dye was then removed by washing with PBS by ultracentrifugation, using a bench‐top ultracentrifuge equipped with a TLA‐55 rotor (both from Beckman Coulter).

EVs were analysed on a NanoAnalyzer N30 instrument (nanoFCM INC), a Nanoflow cytometer dedicated instrument. EV samples were diluted in PBS prior to analysis to allow for 2,000—12,000 particle counts to be recorded during the analysis. Device setup was as previously detailed in Tian et al. (2018), and maintained for all EV samples and standards. Briefly, particle signal acquisition was performed for 1 min using a 488 nm blue laser set to 10 mW and/or a 638 nm red laser set to 20 mW and 10% SS decay, at a sampling pressure of 1.0 kPa modulated and maintained by an air‐based pressure module. Light scattering and fluorescence of individual EVs were collected on single‐photon counting avalanche photodiodes detectors on three channels: side scatter (SSC) FF01 ‐ 488/24 (trigger channel), 524/20 and 670/30 bandpass filters. Sample fluid was focused to ∼1.4 μm using HPLC‐grade water as the sheath fluid via gravity feed. Optical alignment was tested and calibrated using fluorescent 250 nm silica nanoparticles. Further calibration measurements were taken prior to analysis using 250 nm silica nanoparticles of known concentration (for EV concentration calculation), and the proprietary four‐modal silica nanosphere cocktail generated by NanoFCM containing nanosphere populations of 68, 91, 113 and 155 nm diameter (for EV size calculation). PBS was used to define the event triggering threshold. Data was generated through the NanoFCM Professional Suite v1.8 software. EV size and concentration were determined by interpolation from the standard curves calculated with the silica nanoparticles standards. PBS was used for background correction of all data presented, and EVs from non‐transfected Expi293F cells were used as fluorescence negative control.

### 2.11 EV analysis by ExoView

EVs were diluted in Solution A (a proprietary formulation containing Tween‐20) and 35 μl of the sample were incubated for 16 h at RT on ExoView Tetraspanin chips (EV‐TC‐TTS‐01), placed in a sealed 24‐well plate. The chips contained spots printed with anti‐CD63 (clone H5C6), anti‐CD81 (clone JS81), or anti‐CD9 antibodies (clone H19a) for EV populations characterisation, or mouse IgG1κ matching isotype antibody, used as a control for non‐specific EV binding. Chips were then washed three times in 1 ml Solution A for 3 min, under gentle shaking, followed by incubation for 1 h at RT with ExoView Tetraspanin Labelling antibodies mix (EV‐TC‐AB‐01), containing CF647‐conjugated anti‐CD63, anti‐CD81 and anti‐CD9 antibodies (same clones as described above), and CF555‐conjugated anti‐GFP antibody (clone 454505R), all diluted 1:5,000 in Solution A with BSA 2%. The chips were then washed once in Solution A and three times in Solution B, followed by a rinse in filtered deionized water and dried. Solution A and B were provided with the ExoView Tetraspanin chips kit (EV‐TC‐TTS‐01). Chips were then imaged with the ExoView R100 reader using the ExoScan 2.5.5 acquisition software. Images acquired were analysed using ExoViewer 2.5.0 software, with EV sizing thresholds set to 50–200 nm diameter. The signal recorded on isotype‐printed spots was used as background control. GFP detection was performed using the CF555‐conjugated anti‐GFP antibody, instead of its intrinsic fluorescence, due to a higher signal‐to‐noise ratio. The percentage of GFP^+^ EVs in each anti‐tetraspanin and anti‐GFP spot was calculated as the ratio of CF555‐positive events to the CF647‐positive events x 100. GFP Mean Fluorescent Intensity was defined as the mean of the CF555 fluorescence intensities detected in each anti‐tetraspanin spot.

### 2.12 EV analysis by single‐molecule localization microscopy

EVs from transfected and control cells were serially diluted in PBS and adjusted to the same final particle count (1.6 × 10^8^ particles), as determined by Nanoparticle Tracking Analysis (NTA) before being sedimented on microscopy‐compatible 384‐well glass plates (Cellvis). Samples were imaged at multiple dilutions using a 60x oil immersion objective (NA = 1.49) mounted on a Ti Eclipse inverted microscope (Nikon), equipped with a FITC filter cube for GFP fluorescence imaging, CoolLED pE4000 illumination system (490 nm illumination) and an Orca Flash 4.0 CMOS camera (Hamamatsu). Multiple images (200×200 μm^2^) were acquired *per* sample using identical illumination (100 ms exposure time) and focus (via Perfect Focus System). To enhance signal‐to‐background, images were averaged using four consecutive frames. Analysed images had an EV density of < 1 EV/10 μm^2^ to avoid particle overlap, and were 65×65 μm^2^ regions cropped at the centre of the image to minimize the effect of vignetting and uneven illumination. EVs were detected as diffraction‐limited objects corresponding to the point spread function of the microscope, confirming the detection of individual vesicles. Particle identification and quantification of peak intensity (ImageJ) were done using a threshold image (> 2‐fold background intensity). Detected spots with areas below 0.1 μm^2^ and above 10 μm^2^ were removed to filter out the noise and larger aggregates. EV samples from non‐transfected Expi293F cells were used as a negative control for GFP fluorescence background.

For determination of single‐molecule GFP copy number, recombinant GFP (rGFP; SinoBiological) was used as reference fluorophore. Briefly, rGFP was added to empty wells in 10‐fold dilutions (from 1 nM) and the multiple dilutions were imaged using identical settings as above. Identification and quantification of peak intensity by ImageJ1.52p from individual fluorophores was achieved using a threshold image (> 2‐fold background intensity). Subsequent time‐resolved imaging of the individual fluorophores was used to confirm the signal originated from single fluorophores, by recording the bleaching traces. Any objects that showed multistep bleaching traces were removed from the analysis. The resulting intensity histogram was used to estimate the average signal of individual fluorophores. Similarly, the intensity histogram of the EVs was used to estimate the average signal and, by directly dividing by the fluorophore intensity, the absolute protein copy number could be estimated. Due to the high heterogeneity and lower purity of the EV samples compared to recombinant GFP samples, a more stringent threshold of three GFP molecules was defined as the detection limit of SMLM in our work to provide for a more robust analysis.

### 2.13 Statistical analysis

Data statistical analysis was performed using GraphPad Prism v.8.0.1. Data was not found to follow a normal distribution by D'Agostino‐Pearson omnibus normality test, therefore, non‐parametric statistical tests were used for the calculation of statistical significance. For comparison of multiple groups, Kruskal‐Wallis non‐parametric test was used, followed by Dunn's *post‐hoc* test. For comparison of EV size distribution profile, a Two‐way ANOVA statistical test was performed, followed by Tukey *post‐hoc* test. Correlation of data obtained by the different EVs analysis techniques was calculated by Pearson correlation analysis. Statistical significance was considered for α = 0.05 and all statistical differences are indicated in the graphs.

### 2.14 Data availability

All relevant EV‐related methods and data for this work were deposited in the EV‐TRACK knowledgebase (EV‐TRACK ID: EV210126) (Deun et al., 2017).

## 3 RESULTS

### 3.1 EV secretion is impacted by overexpression of specific EV‐sorting proteins

**FIGURE 1:**
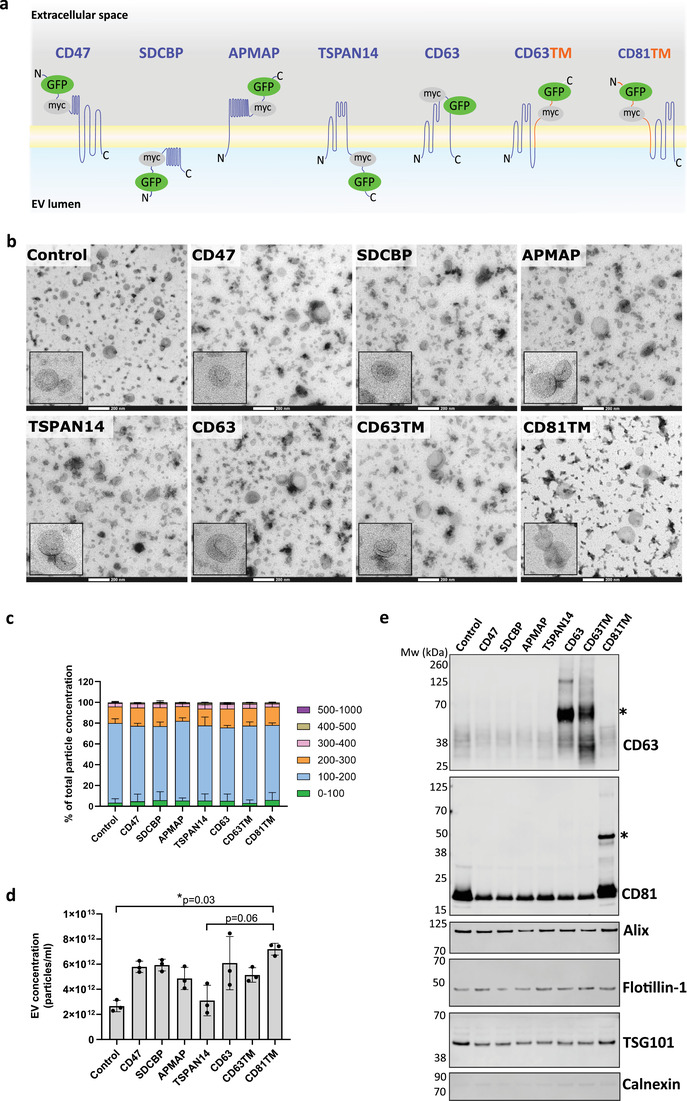
Characterisation of Extracellular Vesicles engineered with proteins for cargo sorting. Candidate proteins present in Extracellular Vesicles (EVs) were selected for engineering by fusion to GFP for cargo loading into the EVs. (a) Schematic representation of the arrangement at the EV membrane of the selected EV‐sorting proteins fused to the model cargo protein GFP. For cargo display at the EV surface, myc tag/GFP were directly fused to extravesicular domains (N‐ or C‐terminal with N‐out/C‐out topology, or extravesicular loops) of EV proteins (purple ribbon), or to the extravesicular PDGFRβ transmembrane domain (TM; orange ribbon) fused to EV proteins with a N‐in/C‐in topology. For intraluminal display of cargo, myc tag/GFP were fused to an intraluminal membrane‐associated EV protein or a N‐in/C‐in EV protein. (b) Representative transmission electron microscopy micrographs of a negative stain of the Expi293F‐derived EVs isolated, showing vesicles with a cup‐shaped morphology. Zoomed‐in insets show the size and morphology of representative EVs. Scale bar: 200 nm. (c) Size distribution of the isolated engineered EVs as analysed by Nanoparticle Tracking Analysis (NTA). Results are represented as the percentage of total EV concentration for each size category indicated. (d) NTA analysis of the concentration of EVs secreted by cells engineered with the various sorting domains. *P*‐values determined by Kruskal‐Wallis statistical test, followed by Dunn's correction for multiple comparisons, are indicated. (e) Representative Western blots of the engineered EVs, showing the presence of protein markers commonly associated with small EVs subpopulations, and reduced levels of the endoplasmic reticulum marker calnexin. The same number of EVs were loaded *per* lane (1.7 × 10^10^ particles). Molecular weight of protein standard is indicated to the left of the blots, and proteins probed to the right. *indicates GFP fusion proteins with CD63 or CD81. All graphs represent mean ± standard deviation. *N* = 3 biological replicates

We used an engineering approach to target the model cargo protein GFP into EVs by overexpression of engineered fusion proteins in producer cells. These engineered proteins were constituted by an anchor EV‐sorting protein with the potential to promote selective cargo enrichment in EVs, fused to myc tag and GFP (Figure 1a; Table S1). The EV‐sorting proteins were selected based on their: (i) high abundance in EVs as determined by the number of reports registered in the databases Vesiclepedia, ExoCarta and EVpedia; (ii) localization at the vesicles membrane; (iii) small size (< 50 kDa) to facilitate overexpression/engineering; (iv) low toxicity and minimal impact on vesicles biogenesis and secretion upon overexpression; and (v) stability and reported tolerance to fusion with other proteins. Selected EV‐sorting proteins were CD47, syntenin‐1 (SDCBP), adipocyte plasma membrane‐associated protein (APMAP), tetraspanin 14 (TSPAN14), CD63 and CD81. GFP was fused to the N‐terminus (N) of CD47 and SDCBP, to the C‐terminus (C) of APMAP and TSPAN14, and into the large loop of CD63 (Figure 1a). Based on the reported membrane topology of these proteins, the N‐out and C‐out disposition of CD47 and APMAP, respectively, as well as the extravesicular localization of the large loop of CD63, allow the display of GFP at the EVs surface. Conversely, the C‐in arrangement of TSPAN14 and the association of SDCBP with the EV membrane would place GFP into the EV lumen. CD63 and CD81 were also fused at their C‐terminus or N‐terminus, respectively, with the transmembrane helix of PDGFRβ, to which GFP was fused for display at EV surface whilst keeping the native N‐in/C‐in arrangement of tetraspanins on the membrane (Figure 1a). PDGFRβ alone was not explored as an EV‐sorting protein due to its reported low abundance in EVs ( (Vallabhaneni et al., 2014), Vesiclepedia, ExoCarta and EVpedia).

Expi293F cells were transiently transfected with DNA vectors encoding the different GFP fusion proteins for the production of the engineered EVs. Cell viability after transfection was comparable to non‐transfected control cells regardless of the fusion protein expressed, and greater than 84% for all conditions tested (Figure S1a). This is indicative of the low predicted cytotoxicity of these constructs and the expressed proteins, minimizing the likelihood of apoptotic bodies secretion to the cell‐conditioned media. EVs were isolated by differential ultracentrifugation and further characterised. TEM imaging of the isolated EVs showed heterogeneous vesicle populations with a lipid membrane, shaped into a round cup‐like morphology and with a small diameter in all conditions (Figure 1b). Size distribution characterisation of engineered EVs by NTA showed the presence of vesicles with an average diameter of 171±12 nm (mean±SD), similar for all conditions analysed including the non‐transfected control (Figure 1c). Interestingly, EV concentration ranged from 2 × 10^12^ to 8 × 10^12^ particles/ml, with a 2–4 fold increase of EV secretion by overexpression of the EV‐sorting proteins CD47, SDCBP, APMAP, CD63, CD63TM and CD81TM, which was statistically significant for CD81TM (Figure 1d), without overall changes in size distribution (Figure 1c).

The EV isolates were also tested by Western blotting for the presence of protein markers characteristic of vesicles of endosomal origin (Figure 1e). Probing for the EV markers CD63 and CD81 led to the detection of the endogenous proteins, but also of the bioengineered proteins for the conditions CD63, CD63TM and CD81TM, with a shifted size corresponding to the fusion with GFP. Higher levels of non‐fused CD63 were detected upon the overexpression of CD63TM‐GFP compared to all other conditions. It is not clear if this corresponds to an increased expression of endogenous CD63 or is the result of CD63 cleavage from the fusion protein. The levels detected for Alix and Flotillin‐1 were comparable across different samples, however, CD81 and TSG101 were slightly reduced upon overexpression of EV‐sorting proteins other than CD81TM, compared to control. Importantly, the endoplasmic reticulum protein marker Calnexin was virtually undetectable on the EV samples analysed, confirming the absence of contaminants such as apoptotic bodies and microsomes. Further analysis of EV samples revealed a high enrichment of CD63, CD81, Alix and Flotillin‐1 in EVs relative to lysates of their parental cells, accompanied by a marked depletion of Calnexin (Figure S1b). Standard analysis using Western blotting, NTA and TEM indicated that cells produced EVs with similar characteristics for all fusion proteins explored, with some small variations observed in the number of EVs secreted and in their tetraspanins composition upon overexpression of specific EV‐sorting proteins.

### 3.2 Efficiency of GFP loading into EVs is dependent upon the EV‐sorting proteins

EV‐sorting proteins were next evaluated for their efficiency in promoting GFP cargo loading into the isolated engineered EVs. First, we evaluated cell transfection efficiency by flow cytometry, with an average of ∼60% of the cells being GFP^+^ for all the EV‐sorting proteins expressed (Figure S1c). GFP levels in Expi293F cells were then confirmed by whole‐cell live imaging, which revealed GFP localization in cell cytoplasm for all the EV‐sorting proteins overexpressed, but also distributed in the plasma membrane for CD63 and CD81TM (Figure S1d). In addition, fluorescence intensity varied across the different fusion proteins and correlated with overexpression level, as confirmed by Western blotting analysis of GFP levels in Expi293F cell lysates (Figure S1d, e).

**FIGURE 2:**
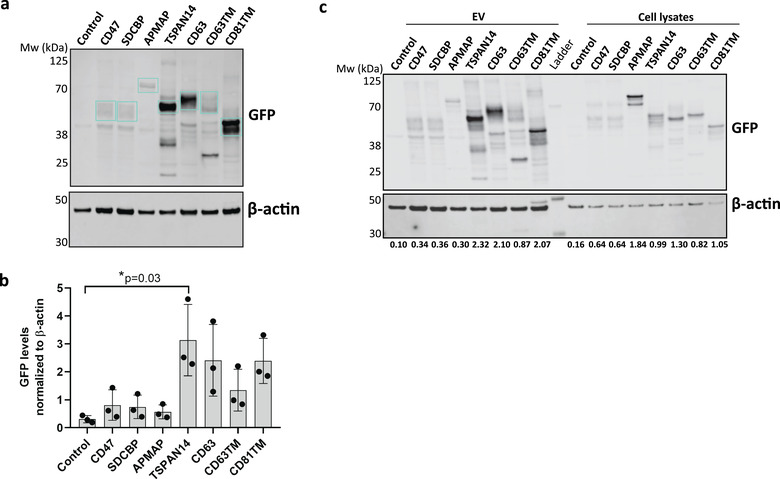
GFP loading efficiency into Extracellular Vesicles is dependent upon the vesicle‐sorting proteins. Expi293F cells were engineered to overexpress the indicated sorting proteins for GFP cargo loading into EVs. (a) Representative Western blot of GFP and β‐actin levels in the whole EV population isolated at 48 h after cell transfection, showing the EV‐sorting proteins tested have a distinct ability in promoting GFP loading into EVs. Molecular weight of protein standard is indicated on the left. Specific bands corresponding to the predicted molecular weight of the fusion proteins are indicated by the blue boxes. (b) Quantification of GFP levels in EVs, normalized to the levels of β‐actin. Quantification of the specific bands corresponding to the fusion proteins was performed by image analysis of the blots using ImageJ software. *P*‐value as determined by Kruskal‐Wallis statistical test, followed by Dunn's correction for multiple comparisons, is indicated. Graph represents mean ± standard deviation. *N* = 3 biological replicates. (c) Representative Western blot for GFP detection in the EVs and whole lysates of corresponding secreting cells, loaded at the same protein amount *per* lane (5 μg). Quantification of GFP levels, normalized to the β‐actin loading control, are indicated below the blot.

GFP levels in EV preparations were analysed by Western blotting at equal particle loading *per* lane to minimize the impact on the analysis of proteins co‐precipitated with EVs, such as the possible presence of soluble GFP in the samples (Corso et al., 2019). Results in Figure 2a show that GFP fusion proteins could be detected in all samples, with high levels for TSPAN14, CD63, and CD81TM. Semi‐quantitative densitometric analysis of the intensity of the specific bands corresponding to GFP fusion proteins further confirmed these results (Figure 2b). TSPAN14 seemed to be the EV‐sorting protein that led to the highest enrichment of GFP in EVs, followed by CD63 and CD81TM, and then by CD63TM, although without statistical significance. CD47, SDCBP and APMAP were the least efficient EV‐sorting proteins in promoting GFP accumulation in EVs. Interestingly, the comparison of GFP levels in Expi293F cell lysates and corresponding EVs, loaded at identical protein amounts in Western blot gels, suggested that GFP cargo fused to most of the EV‐sorting proteins was selectively incorporated into EVs (Figure 2c). The overexpression of TSPAN14, CD63 and CD81TM led to a ∼2‐fold enrichment of GFP fusion proteins in EVs compared to parental cell lysates (Figure 2c). In contrast, APMAP‐GFP fusion was detected at high levels in Expi293F cell lysates, but barely present in EVs (Figure 2c), suggesting the fusion protein is not selectively enriched in the vesicles compared to the parental cells. We also analysed the levels of GFP protein in EVs after overexpression of free, cytosolic GFP in the EV parental cells. Interestingly, the levels of GFP found in EVs passively loaded were similar to those of GFP actively loaded into EVs by TSPAN14 (Figure S1f).

Western blotting analysis of the isolated EVs and their parental cells identified the EV‐sorting proteins that led to the highest enrichment of GFP in EVs in an easy‐to‐implement and practical way. However, it did not provide details on the GFP cargo distribution among different vesicle subpopulations, being incapable of completely distinguishing specific loading into EVs from free soluble protein contaminating the EV samples.

### 3.3 Single‐vesicle analysis reveals EV heterogeneity and incorporation of GFP cargo into distinct subpopulations of engineered EVs

**FIGURE 3:**
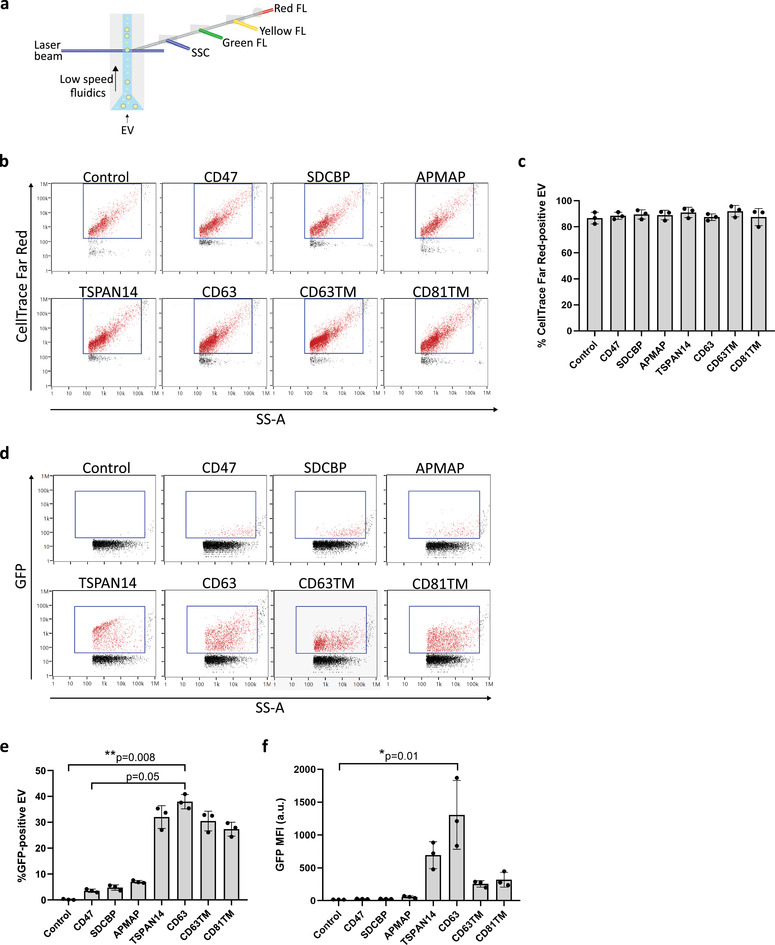
Nanoflow cytometry analysis of Extracellular Vesicles at the single‐vesicle level reveals a heterogeneous distribution of GFP among vesicle subpopulations. (a) Illustration of the operational configuration of a NanoAnalyzer N30 (nanoFCM Inc.) nanoflow cytometer. The slow speed fluidics and the fluorescence‐triggered event detection allow the accurate analysis of small EVs at the single‐vesicle level. SSC: Side scatter; FL: fluorescence. (b) Representative dot plots of Side Scatter (SS‐A) versus CellTrace Far Red dye intensity of gated single vesicles isolated from Expi293F cells engineered with different sorting proteins as indicated, at 48 h post‐transfection. PBS was used as control for EV gating. (c) Percentage of CellTrace Far Red^+^ EVs in the total population of vesicles analysed. (d) Representative dot plots of Side Scatter (SS‐A) versus GFP intensity of gated single vesicles, isolated from Expi293F cells engineered with different sorting proteins as indicated, at 48 h post‐transfection. (e) Percentage of GFP^+^ EVs and (f) mean fluorescence intensity (MFI) of GFP in the total population of vesicles analysed. All graphs represent mean ± standard deviation. *P*‐values as determined by Kruskal‐Wallis statistical test, followed by Dunn's correction for multiple comparisons, are indicated. *N* = 3 biological replicates

To assess GFP cargo distribution among different vesicle subpopulations, isolated EVs were analysed at the single‐vesicle level by Nanoflow cytometry, using a NanoAnalyzer N30 cytometer (nanoFCM INC.). This instrument uses a low‐speed fluidics system, coupled with simultaneous side scattering‐ and fluorescence‐triggered event detection for the specific and sensitive analysis of particles in suspension (Figure 3a). Calibration of the instrument with silica beads allowed an accurate analysis of EVs at the sub‐micron scale, detecting particles down to ∼40 nm in size (Figure S2a). In these conditions, NanoAnalyzer N30 was capable of detecting particles in suspension in EV samples at higher levels than in blank controls (Figure S2b). EVs had an average diameter of 74±2 nm, independently of the sorting protein used for vesicle engineering (Figure S2c). EV concentration was determined to range from 2 × 10^13^ to 7 × 10^13^ particles/ml for the different sorting proteins used, with CD63, CD63TM and CD81TM increasing EV release by 2‐fold (Figure S2d).

Since the nanoflow cytometer NanoAnalyzer N30 can detect any type of small particle in suspension, including secreted proteins and particle aggregates, we further evaluated the purity of our vesicle preparations by labelling the total EVs in our samples with the CellTrace Far Red dye (Figure 3b). Nanoflow cytometry analysis of labelled samples confirmed that on average, more than 85% of the particles in all our samples were EVs (Figure 3b, c). EVs were next analysed for their fluorescent GFP content. Figure 3d shows representative dot plots of GFP fluorescence intensity of gated particles for each EV‐sorting protein. The control sample had no detectable fluorescence. Quantification of GFP^+^ EVs across independent experiments (Figure 3e) showed that GFP cargo could only be detected for up to 50% of the EVs, revealing a marked heterogeneity of the particles. The EV‐sorting proteins TSPAN14, CD63, CD63TM and CD81TM were the most efficient EV‐sorting proteins in promoting GFP loading into EVs, with CD63 allowing enrichment of GFP in a statistically significant higher percentage of vesicles when compared to control. On the other hand, only 3–7% of the EVs engineered with CD47, SDCBP and APMAP proteins were GFP^+^, a percentage similar to that obtained for passive loading of GFP in EVs upon overexpression of free, cytosolic GFP in parental cells (Figure S2e). The mean fluorescence intensity (MFI) of engineered EVs was also analysed as a measurement of GFP enrichment in the vesicles (Figure 3f), with higher MFI corresponding to a higher level of GFP in EVs. Interestingly, MFI analysis revealed the highest GFP levels for EVs engineered with the EV‐sorting protein CD63, followed by TSPAN14, although this difference was not statistically significant. Surprisingly, MFI for EVs engineered with CD63TM and CD81TM was lower, although not significantly, than for CD63‐ and TSPAN14‐EVs, despite the similar percentage of GFP^+^ EVs detected for all these conditions (Figure 3e). These data suggest that CD63TM and CD81TM EV‐sorting proteins can promote GFP cargo loading in a significant fraction of EVs, but at low levels *per* vesicle.

**FIGURE 4:**
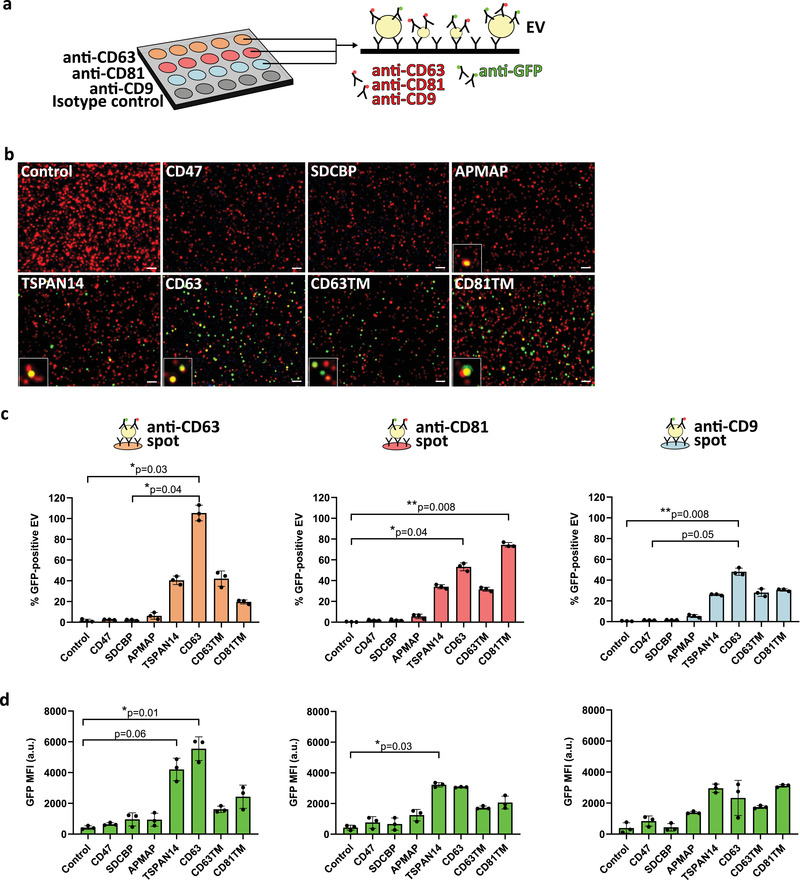
ExoView analysis at the single‐vesicle level of GFP‐engineered Extracellular Vesicles subpopulations. (a) Scheme illustrating the working principle of ExoView chips (NanoView). EV samples were captured on the surface of chips printed with antibodies anti‐tetraspanin CD63 (orange), CD81 (red), and CD9 (blue) at defined spots. Spots with isotype antibodies (grey) were used as control. EVs bound to the anti‐CD63, anti‐CD9 or anti‐CD81 spots were then immunolabeled with CF647‐conjugated anti‐tetraspanins (CD63, CD81 and CD9) antibody mix, plus CF555‐conjugated anti‐GFP antibody. Engineered EVs isolated from Expi293F cell cultures at 48 h after cell transfection were diluted to prevent over‐coverage of the chip, and then incubated overnight on top of the chip surface. (b) Representative micrographs of engineered EVs bound to the anti‐CD9 spot on the ExoView chip. Whole EV populations were detected by immunolabelling with CF647‐anti‐tetraspanins antibodies (red dots), and GFP‐EVs were detected with CF555‐anti‐GFP antibody (green dots). Signal co‐localization appears in yellow and is highlighted in figure insets. Scale bar: 3 μm. (c) Quantification of the percentage of GFP^+^ EVs and (d) the Mean Fluorescence Intensity of CF555 anti‐GFP immunolabelling of the EVs captured in the anti‐tetraspanins spots present on the ExoView chip for each of the EV‐sorting proteins indicated. The percentage of GFP^+^ EVs was calculated as the ratio of CF555‐positive events to the CF647‐positive events x 100. All graphs represent mean ± standard deviation. *P*‐values are indicated, as determined by Kruskal‐Wallis statistical test, followed by Dunn's correction for multiple comparisons. *N* = 3 biological replicates

Isolated EVs were also analysed by ExoView for protein profiling of the different GFP^+^ vesicle subpopulations at the single‐vesicle level. ExoView technology is based on single‐particle interferometric reflectance imaging detection of EVs captured on a layered silicon substrate chip, constituted by an array of spots printed with different antibodies. This can be combined with EV immunostaining with fluorophore‐conjugated antibodies, followed by fluorescence imaging, and multiplexed for the simultaneous detection of up to three distinct fluorophores. In this work, EVs were subjected to standard mild permeabilization as *per* manufacturer recommendation, and captured on the silicon substrate array functionalised with anti‐CD63, anti‐CD81 and anti‐CD9 antibodies in different spots for EV capture (Figure 4a). EVs captured in all spots were simultaneously immunostained with anti‐GFP fluorophore‐conjugated antibodies for GFP detection and quantification at a higher signal‐to‐noise ratio, and with fluorophore‐conjugated anti‐tetraspanins antibodies (CD63, CD81, CD9) for detection of engineered and non‐engineered EV populations (Figure 4a). Mild permeabilization of EVs is necessary for the detection of GFP^+^ EVs carrying intraluminal GFP. Figure 4b represents the detection of GFP‐EVs (green) among the total vesicle population (red) captured on an anti‐CD9 spot, evidencing that GFP‐loaded EVs also carry characteristic EV protein markers (co‐localization in yellow). Spots functionalised with IgG isotype were used as control of non‐specific EV adsorption to the chip substrate, with no significant numbers of particles counted (data not shown). The percentage of GFP^+^ EVs amongst the total vesicles population was then quantified in all anti‐tetraspanin capture spots (Figure 4c). The highest percentage of GFP^+^ EVs was detected upon the overexpression of the EV‐sorting proteins TSPAN14, CD63, CD63TM and CD81TM for the anti‐CD63, ‐CD81 and ‐CD9 capture spots tested. ExoView allowed to further characterise the heterogeneous distribution of GFP in the various EV subpopulations. Upon the overexpression of the CD63‐GFP fusion protein, all the EVs captured on the anti‐CD63 spot were GFP^+^ (Figure 4c, left panel), and more than 50% of the EVs captured on the anti‐CD81 and anti‐CD9 spots were GFP^+^ (Figure 4c, middle and right panel, respectively), with the percentage of GFP^+^ EVs being significantly higher in all spots compared to control. On the other hand, CD63TM and TSPAN14 overexpression led to GFP loading into only 30–40% of the CD63^+^, CD81^+^ and CD9^+^ EVs. Overexpression of CD81TM led to the identification of a significantly higher percentage of CD81^+^GFP^+^ EVs compared to control, with nearly 80% of the EVs carrying GFP. However, only 20% of the CD81TM‐engineered EVs were CD63^+^GFP^+^, and 30% were CD9^+^GFP^+^. Of note, overexpression of CD47, SDCBP and APMAP led to an enrichment of GFP into less than 10% of CD63^+^, CD81^+^ or CD9^+^ vesicles. Interestingly, the same overall results of GFP distribution in EVs upon engineering with the different sorting proteins could be obtained upon direct analysis of EV‐enriched cell‐conditioned media, depleted only of larger particles such as apoptotic bodies and microvesicles (Figure S3a), being indicative of the ability of ExoView to perform EV analysis without prior small EV isolation.

We also analysed the levels of GFP enrichment in the engineered EVs isolated from cell‐conditioned media. For that, the mean intensity of the GFP immunostaining (GFP MFI) in the EVs captured in the anti‐CD63, ‐CD81 and ‐CD9 spots was determined (Figure 4d). Results showed that CD63‐ and TSPAN14‐engineered EVs had the highest levels of GFP relative to control (Figure 4d). Moreover, GFP cargo enrichment was particularly high in the CD63^+^ EV subpopulations (Figure 4d, left panel), followed by the CD81^+^ EVs (Figure 4d, middle panel), albeit no significant differences were observed between the CD63‐ and TSPAN14‐EVs compared to the remaining EV‐sorting proteins. CD81TM was the third‐ranked EV‐sorting protein promoting GFP loading at higher levels into EVs, particularly in CD9^+^ vesicles, although not significantly comparing with control nor the other EV‐sorting proteins. Fluorescence detected in the untransfected control was at an intensity level similar to that registered for the control isotype spot included in ExoView chips, corresponding to background fluorescence (Figure S3b). Importantly, direct analysis of supernatants enriched on EVs depleted of larger particles but without further concentration, also revealed a higher GFP intensity upon overexpression of CD63, TSPAN14 and CD81TM (Figure S3c), confirming these EV‐sorting proteins as very efficient promoters of GFP loading into EVs.

In summary, ExoView allowed the characterisation of heterogeneous EV subpopulations loaded with GFP, estimating also the level of enrichment of the fluorescent cargo in the loaded vesicles. However, sensitivity limitations prevented the determination of the precise amount of GFP molecules carried in the vesicles.

### 3.4 Single‐vesicle analysis at the single‐molecule level reveals differential efficiency of EV‐sorting proteins in promoting GFP cargo loading into EVs

**FIGURE 5:**
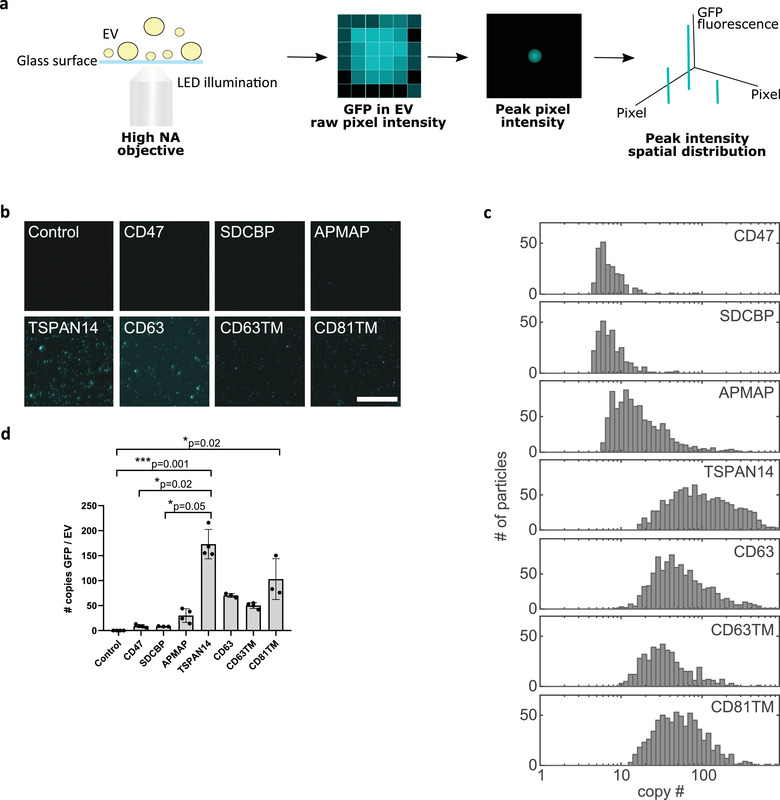
Single‐Molecule Localization Microscopy enables determination of the number of GFP molecules loaded into single vesicles according to their vesicle‐sorting protein. (a) Illustration of the working principle of the Single‐Molecule Localization Microscopy (SMLM) setup used in this work. Engineered small EVs isolated from Expi293F cells‐conditioned media were diluted in PBS and added to a multi‐well plate with a glass bottom, where vesicles deposited. GFP on EVs was excited with a monochromatic LED and the reflected light collected using an objective with a high numerical aperture (NA) for photon gathering maximization. The peak intensity of the point‐spread function of each molecule detected was retrieved, allowing its accurate localization and thus, quantification of the number of molecules present in a vesicle. (b) Representative micrographs of GFP detection on diluted EVs deposited on the glass bottom of the analysis plate for each of the sorting proteins indicated. Scale bar: 30 μm. (c) Distribution profile of GFP copy number in single vesicles engineered with the indicated sorting proteins. (d) Quantification of the average number of GFP molecules present in the single vesicles. Graph represents mean ± standard deviation. *P*‐values are indicated, as determined by Kruskal‐Wallis statistical test, followed by Dunn's correction for multiple comparisons. *N* = 3 or 4 biological replicates

Analysis of engineered EVs at the single‐vesicle level provides important information about cargo distribution on different vesicle subpopulations, but gives limited insights regarding cargo enrichment in each vesicle analysed. To accurately evaluate the efficiency of GFP cargo loading into the engineered EVs at the single‐vesicle and single‐molecule levels, we implemented a workflow using single‐molecule localization microscopy (Figure 5a). Absolute quantification of the copy number of GFP molecules *per* single vesicle was determined using purified recombinant GFP protein as a reference. To achieve this, serial dilutions of GFP protein were imaged to determine optimal fluorophore surface coverage. Subsequent photobleaching imaging confirmed single‐molecule readout, as any objects that showed multistep bleaching traces were removed from analysis (Figure S4a–c). The resulting intensity histogram was used to estimate the average signal of individual fluorophores. Then, EV samples were diluted to prevent overlapping of individual GFP‐loaded EVs at the imaging surface, samples were adsorbed onto a glass bottom of a multi‐well plate and imaged as diffraction‐limited objects (Figure 5b). Only GFP^+^ EVs are analysed by SMLM, whereas, GFP^–^ EVs are not detected, due to the reliance of this technique on fluorescent signal detection.

In a similar analysis as for purified GFP protein, the intensity histogram of the GFP^+^ EVs was used to estimate the average signal and, by directly dividing the value by the fluorophore intensity, the absolute protein copy number could be estimated (Figure 5c, d). In contrast to the other single EV analysis methods described above, SMLM does not suffer from a low limit of detection due to the high numerical aperture (NA) objective used for EVs imaging, which enables high signal‐to‐noise. Analysis of the GFP copy number distribution in GFP^+^ EVs for each protein‐sorting domain confirmed a heterogeneous distribution of cargo across single vesicles (Figure 5c). Overexpression of TSPAN14 resulted in the highest intensity values leading to an average enrichment of 173 molecules *per* EV (Figure 5d). CD81TM, CD63 and CD63TM also promoted significantly higher enrichment of GFP into EVs when compared to control, with average loadings of 103, 70 and 50 GFP copies *per* vesicle, respectively. On the other hand, overexpression of CD47, SDCBP and APMAP generated populations of engineered EVs loaded with more modest levels of GFP (9, 8 and 30 copies *per* vesicle, respectively) suggesting a less efficient EV‐sorting process.

## 4 DISCUSSION

The ability to load EVs with relevant therapeutic molecules and to specifically and efficiently deliver them to target tissues is of great importance for the development of EVs for drug delivery applications. Engineering of EVs to incorporate protein cargo has been explored for these purposes, although with limited success when EVs are administered *in vivo*. Most of the strategies employed for loading therapeutic proteins into EVs are based on cell engineering, with the generation of fusion proteins where the cargo of interest is fused to proteins that are enriched in the vesicles (Yang et al., 2018). In this work, we evaluated the efficacy and efficiency of different EV‐sorting proteins as drivers of protein cargo loading into EVs, expanding the range of sorting proteins that can be successfully used for repurposing EVs as delivery vehicles of protein‐based therapeutics. Furthermore, we established a fast and effective workflow based on transient transfection of EV‐secreting Expi293F cells for the screening of optimal EV‐sorting proteins, an advantage compared to previous works where stably transfected cell models were used (Dooley et al., 2021). In our study, Expi293F cells were selected as the EV cell source as they hold important features for EV production at large‐scale both in a laboratory setting and at a clinical stage. Expi293F suspension cells grow to high cell densities in a chemically defined serum‐free culture medium, requiring fewer media *per* batch of produced EVs. Expi293F cells are also optimized for high‐efficiency transient expression, yielding a high protein and mRNA production with improved cell viability (Fang et al., 2017), which is an advantage for the production of EVs engineered with protein cargo. In addition, Expi293F‐derived EVs were not toxic nor immunogenic when injected *in vivo* in a pre‐clinical mice model (Saleh et al., 2019), rendering Expi293F cells a suitable starting platform for the production of engineered EVs in future clinical translational studies. Furthermore, EVs derived from cell lines of human embryonic kidney background are already under evaluation for safety in human clinical trials (study ID: NCT04747574; ClinicalTrials.gov) (NIH ‐ US, 2021).

In line with recent studies (Corso et al., 2019; Dooley et al., 2021), our strategy of EV engineering focused on the development of protein fusions for GFP loading using as anchors proteins associated with the EV membrane. This included the fusion of the GFP at different regions of the EV transmembrane proteins selected or to snorkel motifs fused to them, to best retain their natural function (Yang et al., 2018). We generated engineered EVs carrying GFP cargo displayed either at the EVs surface or in their lumen. Our results further support the ability to display cargo proteins at the surface of EVs for therapeutic purposes, as GFP can be swapped for cell type specific‐tropic moieties (e.g., peptides, antibodies) to target EVs to tissues or cells of interest (Alvarez‐Erviti et al., 2011; Kooijmans et al., 2016). Likewise, we further demonstrated the ability of loading proteins to the EV lumen, protected from extracellular degradation. Importantly, EV engineering did not alter the main physical properties of the isolated EVs, namely morphology and size, but resulted in some changes of the surface protein composition of the EVs, not only restricted to the presence of the overexpressed fusion protein, but also comprising changes in the levels of other proteins commonly found in EVs. These changes can have more profound repercussions in EVs function, for instance altering their *in vivo* biodistribution, as recently demonstrated by our group (Lázaro‐Ibáñez et al., 2021). In addition, we observed an upregulation of the secretion of EVs from the engineered cells. These observations are consistent with published data supporting that the overexpression of canonical EV proteins often results in changes in the protein content and levels of EV secretion (Böker et al., 2018; Corso et al., 2019; Lázaro‐Ibáñez et al., 2021). Together, these results suggest that the overexpression of EV proteins strongly impacts the EV biogenesis and secretion pathways in Expi293F cells. In this work, we used NTA and Nanoflow cytometry for biophysical analysis of the isolated EVs. The mean EV size estimated by Nanoflow cytometry was smaller than by NTA. These results are possibly related to the different sensitivity of both instruments to detect particles in suspension, reflecting a higher sensitivity of the NanoAnalyzer N30 cytometer to detect smaller vesicles. On the other hand, NTA is not able to accurately detect smaller vesicles nor discriminating variations in their small size, leading to an estimation of a higher average vesicle diameter (Arab et al., 2021; Vogel et al., 2021). Consequently, the differences in the working principle and in the sensitivity of both techniques to detect EVs result in an underestimation of their concentration by NTA when compared to Nanoflow cytometry.

To evaluate the efficiency of the different EV‐sorting domains tested in promoting protein cargo loading into EVs, we performed an extensive characterisation of the isolated EVs using complementary analysis techniques that allowed scrutinization of subpopulations of vesicles from a bulk level to a single‐vesicle and single‐molecule resolution. The importance of the characterisation of isolated EVs at such high resolution is motivated by the increasing recognition of the heterogeneity and complexity of the EV populations secreted by cells (Kalluri & LeBleu, 2020). The complexity of secreted EVs stems from their origin through diverse biogenesis pathways in the cell, which result in EVs with similar physical properties but distinct cargo composition (van Niel et al., 2018). For instance, preparations of small‐sized EVs may comprise cell membrane‐derived ARRDC1‐mediated microvesicles as well as multivesicular bodies (MVB)‐derived particles. Also, EVs originated in MVBs may be formed by ESCRT‐dependent or ‐independent mechanisms, but how the different EV biogenesis routes impact the cargo loaded into EVs is still largely unknown. A detailed characterisation of the isolated EVs at the single‐vesicle level can contribute to further unravel these mechanisms, allowing the implementation of strategies for the isolation of increasingly pure EV subpopulations with enhanced therapeutic potential. According to our results, Western blotting, Nanoflow cytometry, ExoView and SMLM were able to detect GFP cargo loading into EVs upon its fusion with all the different EV‐sorting proteins tested. Although Western blotting is the most commonly used technique for analysis of the protein composition of EVs, this technique is based on bulk analysis of the total protein content of samples and, therefore, it cannot distinguish between cargo specifically loaded into EVs and soluble contaminants present in the preparation, neither differential levels of cargo loaded across distinct EV subpopulations. Moreover, the technique is only semi‐quantitative, being highly dependent on the specificity and affinity of the antibody‐antigen interaction, and requiring extensive validation of the reagents used, such as, recombinant protein controls used as external calibrators for calculation of standard curves for interpolation of the results. This technique also suffers from the lack of reliable endogenous EV proteins that can be used as a reference protein for normalization of results. Therefore, the reliable quantification of GFP‐loaded EVs and the analysis of the GFP cargo copy number loaded in single EVs requires high‐resolution techniques. Indeed, analysis of the same EVs by Nanoflow cytometry, ExoView and SMLM revealed that GFP cargo was not distributed homogeneously amongst EV subsets from the same preparations. In the work of Corso *et al*. (Corso et al., 2019), similar approaches for orthogonal EV analysis were evaluated, including characterisation of EV preparations in bulk using Western blotting, at the single‐vesicle level using imaging flow cytometry and at the single‐molecule level using fluorescence correlation spectroscopy. Their results are in line with ours, revealing a heterogeneous distribution of GFP cargo across EV subpopulations. Because Nanoflow cytometry and ExoView were developed for the specific analysis of nanoparticles, our approach of single‐vesicle characterisation by these techniques is likely more easily implementable than an imaging flow cytometry workflow, requiring less methodological optimizations for accurate EV characterisation (Görgens et al., 2019), particularly if a multiplexed analysis of EVs to further detail their identity is intended. On the other hand, for the single‐molecule analysis of EVs, fluorescence correlation microscopy might have increased accuracy in distinguishing EV‐associated from non‐vesicular GFP.

Significant efforts have been made to develop Nanoflow cytometry for the reliable analysis of submicron‐sized particles. Analysis of EVs by conventional flow cytometry requires their coupling to larger beads, but Nanoflow cytometry allows direct characterisation of single vesicles in suspension. Nanoflow cytometry analysis of the isolated EVs detected GFP in only a proportion of the particles analysed, despite the high purity of the EV preparations, suggesting that a significant fraction of the EVs detected were generated through alternative biogenesis routes not involving the designed EV‐sorting fusion proteins (Lázaro‐Ibáñez et al., 2021; Leidal & Debnath, 2020). In agreement with our findings, previous studies using Nanoflow and flow cytometry have also reported heterogeneous loading of GFP cargo across subpopulations of engineered EVs, detecting percentages of GFP^+^ EVs in the same range as in our study for similar EV‐sorting proteins (Corso et al., 2019; Dooley et al., 2021; Görgens et al., 2019). Furthermore, the multiparametric analysis of EVs by Nanoflow cytometry demonstrated that the overexpression of free, cytosolic GFP in Expi293F cells is not sufficient to allow active loading of the protein into EVs, being far less efficient compared to some of the EV‐sorting proteins evaluated in this work, a relevant feature that could not be distinguished in EV bulk analysis by Western blotting. Importantly, the effective detection of fluorochromes as a single event in flow cytometry instruments requires the presence of a minimal number of fluorescent molecules, usually reported as the MESF (Molecules of Equivalent Soluble Fluorophore) value (Schwartz et al., 2002). It is possible that GFP^dim^ vesicles present in the EV preparations that carry a number of GFP molecules below MESF, do not have a fluorescence intensity above the threshold set with control EVs to define the GFP^+^ and GFP^–^ EV populations, contributing to the reduced percentage of GFP^+^ EVs detected in the analysis. In fact, the GFP copy number distribution *per* vesicle determined by SMLM shows the presence of engineered EVs carrying down to 10 GFP molecules across different conditions, particularly when the fluorescent protein was fused to CD47, SDCBP and APMAP, which directly correlates with the low GFP MFI and percentage of GFP^+^ EVs detected for these conditions by Nanoflow cytometry. Likewise, the low GFP MFI values obtained by Nanoflow cytometry for EVs engineered with CD63TM and CD81TM in comparison with CD63 and TSPAN14, despite the percentage of GFP^+^ EVs being similar in all these conditions, is explained by the GFP copy number distribution *per* vesicle, as determined by SMLM. Indeed, overexpression of CD63TM and CD81TM seems to originate a larger subpopulation of GFP^dim^ EVs carrying low levels of GFP.

The distribution of GFP in different subpopulations of engineered EVs was also analysed at the single‐vesicle level by ExoView. This technique based on capturing EVs onto chips spotted with antibodies allows a fast and multi‐parametric analysis of the vesicles without prior time‐consuming sample preparation procedures. In this work, ExoView chips contained antibodies recognizing the canonical tetraspanin markers CD63, CD81 and CD9, commonly assigned to EVs from endosomal origin, allowing the profiling of the tetraspanins composition of the GFP^+^ EVs. Overexpression of CD63 and CD81 led to the detection of very high percentages of GFP^+^ EVs, even implying that most of the detected EVs were successfully engineered. However, these results should be interpreted carefully, as they might result from bias in the capture of the EVs in the spots printed with the antibody recognizing the tetraspanins being overexpressed, driven by the high antibody‐antigen affinity. Our findings revealed that GFP cargo is equally distributed among CD63^+^ and CD81^+^ EVs subpopulations, with the percentage of GFP^+^CD63^+^ EVs being similar to the percentage of GFP^+^CD81^+^ EVs for all conditions analysed, except those where these tetraspanins were overexpressed. Similarly to Nanoflow cytometry, quantification of GFP MFI of the EVs captured in anti‐tetraspanin spots provided an approximation of the relative GFP enrichment in the vesicles upon overexpression of the different EV‐sorting proteins. CD63 and TSPAN14 were the EV‐sorting proteins that promoted the highest levels of GFP loading into the captured EVs, in accordance with the findings from EV analysis by Nanoflow cytometry. Interestingly, the highest GFP MFI levels were detected for the CD63^+^ EV subpopulations, suggesting an efficient GFP cargo loading into EVs originated from biogenesis pathways involving this tetraspanin (Hurwitz et al., 2016). The detection of GFP with CF555‐conjugated anti‐GFP antibodies improved the reproducibility of the ExoView assay comparing with direct GFP fluorescence measurement due to enhanced stability of the CF555 fluorophore. This approach could easily detect the GFP displayed at the surface of EVs upon fusion to the EV‐sorting proteins CD47, APMAP, CD63, CD63TM and CD81TM. Conversely, the detection of GFP loaded in the EV lumen by SDCBP and TSPAN14 was only possible upon the permeabilization of the vesicles with the buffers used in ExoView assay standard conditions containing low detergent concentrations. Nonetheless, we cannot exclude the possibility that EVs could have been partially degraded or their membrane disrupted, during isolation, handling and storage (Cvjetkovic et al., 2016; Nordin et al., 2015; Osteikoetxea et al., 2015), leading to intraluminal GFP exposure for detection in vesicle membrane fragments. In fact, this limitation precluded the reliable analysis of the samples by direct capture of EVs with intraluminal GFP in the ExoView chips using immobilized anti‐GFP antibodies.

We further analysed the engineered EVs at the single‐molecule level, using a simplified setup of SMLM workflow to quantify the copy number of GFP protein cargo loaded into the vesicles. In a previous study, a similar analysis of protein cargo copy number loaded into EVs was performed using fluorescence correlation spectroscopy (Corso et al., 2019). Here, we implemented a single‐molecule imaging technique based on photobleaching events of single molecules, and developed an easy‐implementable image analysis approach for copy number calculation. Importantly, SMLM provides accurate EV analysis also for particles carrying down to three GFP molecules. Using SMLM, we verified that the most efficient EV‐sorting proteins led to an enrichment of GFP from 50 to ∼170 molecules *per* vesicle. GFP fusion to the C‐terminus or the large extravesicular loop of CD63 led to a loading of a number of cargo molecules *per* vesicle similar to previously reported (Corso et al., 2019). However, TSPAN14 and CD81TM fusion proteins were more efficient in enriching GFP cargo in EVs than other types of EV‐sorting proteins previously described (Corso et al., 2019). Of note, these results cannot be directly compared as the cell lines and expression vectors used in this study are different and could influence the expression levels of GFP in the cells and consequently in the vesicles they secreted.

**FIGURE 6:**
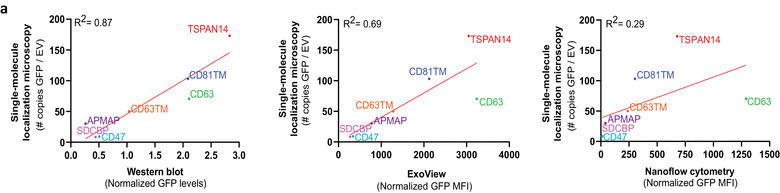
Accurate characterisation of Extracellular Vesicles subpopulations at the single‐vesicle and single‐molecule level is dependent on the technical approach used for vesicles analysis. (a) Correlation analysis of GFP levels in small EVs derived from engineered Expi293F cells, as determined by Single‐Molecule Localization Microscopy (SMLM) versus Western blotting (left panel), SMLM versus ExoView (middle panel) and SMLM versus NanoAnalyzer N30 Nanoflow cytometry (right panel). For the ExoView dataset, the average of GFP MFI measured on the anti‐CD63, ‐CD81 and ‐CD9 spots was considered. Labels in the graphs indicate the sorting protein used for EVs engineering. All datasets were normalized to the untransfected control condition. R^2^ values for the Pearson correlation between SMLM and the different analysis techniques are also indicated in the graphs

From the techniques explored in this work to quantify the efficiency of the EV‐sorting proteins in promoting cargo loading into EVs, SMLM was the most sensitive and accurate, characterising GFP loading down to a single‐molecule level without relying on prior detection, gating or capturing of the vesicles themselves. For this reason, we considered SMLM as the reference method for comparison of the performance of the other techniques tested in this work. Data correlation analysis revealed that GFP quantification by Western blotting highly correlated with SMLM quantification, followed by ExoView and Nanoflow cytometry (Figure 6a). This derives from the principle of GFP analysis by the different techniques: both Western blotting and SMLM detect only GFP and GFP^+^ EVs, respectively, whereas Nanoflow cytometry and ExoView allow the detection of both GFP^–^ and GFP^+^ EVs, with MFI being calculated in this work for the whole EV population detected. Nonetheless, we observed that all techniques consistently identified TSPAN14, CD63 and CD81TM as top candidates for loading of GFP into EVs, followed by CD63TM. Previous works had also identified tetraspanins (namely CD63 and CD81) as promising EV‐sorting proteins able to promote the enrichment of GFP cargo in moderate‐to‐high levels in a significant percentage of secreted EVs (Corso et al., 2019; Dooley et al., 2021). The selection of the most suitable techniques for characterisation of engineered EVs will always depend on the purpose of the analysis and will rely on the advantages and disadvantages of each technique, as summarized in Table 1. Western blotting might be sufficient for a fast screening to identify cargo loaded into EVs, but its reliability is limited, particularly for EV samples with lower purity, where contaminant cargo outside EVs is also detected. Moreover, it is not quantitative neither allows an accurate determination of the efficiency of cargo loading (calculated as the percentage of GFP^+^ vesicles). This information is particularly important for a proper estimation of the EV doses that need to be used to achieve functional delivery of protein cargo both for *in vitro* and *in vivo* treatments, especially if only a small portion of the vesicles carry adequate levels of the cargo of interest. On the other hand, Nanoflow cytometry and ExoView were able to capture the heterogeneous distribution of GFP cargo across different subpopulations of vesicles, although with less accurate quantification, mainly due to the sensitivity for minimal GFP fluorescence detection and dependence on capturing and detection antibodies for EVs analysis, respectively. Despite the advantages of SMLM for quantification of cargo loading into EVs, it is not suitable for the determination of EV size and concentration, and the calculation of EV engineering efficiency is only possible upon whole EV population labelling, for instance with a membrane‐intercalating dye (Lázaro‐Ibáñez et al., 2021).

**TABLE 1:** Advantages and disadvantages of Western blotting, Nanoparticle Tracking Analysis, Nanoflow cytometry, ExoView and SMLM for the analysis of heterogeneous engineered EV subpopulations at the single‐vesicle and single‐molecule level

	Western blotting	Nanoparticle tracking analysis	Nanoflow cytometry	ExoView	SMLM
Measurement of EV size and concentration	**−**	**++** (> 50‐1,000 nm)^a^ ^,^ ^b^	**++** (40‐200 nm)^a^ ^,^ ^c^	**+** (50‐200 nm)^a^ ^,^ ^d^	**−**
EV detection sensitivity and resolution	**−** (bulk analysis only)	**+** (single vesicle)	**++** (single vesicle)	**++** (single vesicle)	**+++** (single vesicle, single molecule)
Analysis of EV populations heterogeneity	**−**	**+**	**++** ^e^	**++** ^e^	**++**
Cargo copy number quantification	**−**	**−**	**−**	**−**	**++**
Fluorescence detection limit	**−**	**−** ^f^	**−** ^g^	**−** (3 CF555 MESF)	**++** (> 1 GFP MESF)
Technology accessibility	**++**	**+**	**−**	**−**	**−**
Expert use	**−**	**+**	**++**	**+**	**++**

− and + signals rank the performance of each technique to the parameters specified. Where applicable, parameter details are indicated between brackets.

^a^ Optimal size range of analysis.

^b^ Optimal working sample dilution for analysis: 10^8^–10^9^ particles/ml.

^c^ Optimal working sample dilution for analysis: 10^8^–10^9^ particles/ml.

^d^ Optimal working sample dilution for analysis: 10^9^–10^10^ particles/ml. Dependent on antibodies printed on the chip and on EVs surface protein composition.

^e^ Multiparametric analysis capability not fully explored in this work.

^f^ Fluorescence mode could not be applied in this work.

^g^ Could not be experimentally determined in this work.

In conclusion, our study demonstrates that engineered EVs have a heterogeneous distribution of cargo across vesicle subpopulations, which can only be characterised upon EV analysis at a single‐vesicle, single‐molecule level, using high‐resolution techniques. These allow the determination of the efficiency of novel EV‐sorting proteins in promoting target cargo loading into vesicles, ultimately providing useful insights on EV biogenesis, loading mechanisms and targeting pathways that have relevant implications for drug delivery, and on dose‐efficacy relationship upon cargo delivery across *in vitro* and *in vivo* studies. We validated SMLM as a very accurate technique for analysis of engineered EVs and proposed novel EV‐sorting proteins that lead to high enrichment levels of target protein cargoes into vesicles. This knowledge can be further applied in future studies exploring the use of EVs as delivery vehicles for more complex therapeutic proteins.

## DISCLOSURE OF INTEREST

Andreia M. Silva, Elisa Lázaro‐Ibáñez, Anders Gunnarsson, Xabier Osteikoetxea, Nikki Salmond, Kristina Pagh Friis, Olga Shatnyeva and Niek Dekker are or were at the time of work completion employees of AstraZeneca R&D. Ben Peacock is an employee of NanoFCM and his contribution to this work was made as part of his employment. Aditya Dhande and George Daaboul are employed by NanoView Biosciences.

## Supporting information

Supporting information.
