# Supplementary material for: Quantification of protein cargo loading into engineered extracellular vesicles at single‐vesicle and single‐molecule resolution
Source: J Extracell Vesicles. 2021 Aug 2;10(10):e12130. doi: 10.1002/jev2.12130 (PMC8329990; doi:10.1002/jev2.12130)
Supplement: Supplementary file 1 — Supporting information. [file JEV2-10-e12130-s001.pdf]

Supplementary materials

Quantification of protein cargo loading into engineered Extracellular Vesicles at single-vesicle and single-molecule resolution.

Andreia M. Silva, Elisa Lázaro-Ibáñez, Anders Gunnarsson, Aditya Dhande, George Daaboul, Ben Peacock, Xabier Osteikoetxea, Nikki Salmond, Kristina Pagh Friis, Olga Shatnyeva & Niek Dekker

Supplementary Table 1

Supplementary Table 1. Protein sequence of the fusion proteins generated with different EV-sorting proteins fused to GFP cargo. Predicted molecular weights of the fusion proteins are indicated in brackets

EV-sorting protein + GFP	Protein sequence
**GFP-CD47**<br>(Mw: 69 kDa)	MPLLLLLPLLWAGALAMVSKGEELFTGVVPILVELDGDVNGH<br>KFSVSGEGEGDATYGKLTLKFICTTGKLPVPWPTLVTTLTYG<br>VQCFSRYPDHMKQHDFFKSAMPEGYVQERTIFFKDDGNYK<br>TRAEVKFEGDTLVNRIELKGIDFKEDGNILGHKLEYNYNSHNV<br>YIMADKQKNGIKVNFKIRHNIEDGSVQLADHYQQNTPIGDGP<br>VLLPDNHYLSTQSALSKDPNEKRDHMVLLEFVTAAGITLGMD<br>ELYKGGAGGNSRPLEPLELGGGGSEQKLISEEDLGGGGSM<br>WPLVAALLLGSACCGSAQLLFNKTKSVEFTFCNDTVVIPCFV<br>TNMEAQNTTEVYVKWKFKGRDIYTFDGALNKSTVPTDFSSA<br>KIEVSQLLKGDASLKMDKSDAVSHTGNYTCEVTELTREGETII<br>ELKYRVVSWFSPNENILIVIFPIFAILFWGQFGIKTLKYRSGGM<br>DEKTIALLVAGLVITVIVIVGAILFVPGEYSLKNATGLGLIVTST<br>GILILLHYYVFSTAIGLTSFVIAILVIQVIAYILAVVGLSLCIAACIP<br>MHGPLLISGLSILALAQLLGLVYMKFVASNQKTIQPPRKAVEE<br>PLNAFKESKGMMNDE**
**GFP-SDCBP**<br>(Mw: 65 kDa)	MPLLLLLPLLWAGALAMVSKGEELFTGVVPILVELDGDVNGH<br>KFSVSGEGEGDATYGKLTLKFICTTGKLPVPWPTLVTTLTYG<br>VQCFSRYPDHMKQHDFFKSAMPEGYVQERTIFFKDDGNYK<br>TRAEVKFEGDTLVNRIELKGIDFKEDGNILGHKLEYNYNSHNV<br>YIMADKQKNGIKVNFKIRHNIEDGSVQLADHYQQNTPIGDGP<br>VLLPDNHYLSTQSALSKDPNEKRDHMVLLEFVTAAGITLGMD<br>ELYKGGAGGNSRPLEPLELGGGGSEQKLISEEDLGGGGSM<br>SLYPSLEDLKVDKVIQAQTAFSANPANPAILSEASAPIPHDGN<br>LYPRLYPELSQYMGLSLNEEEIRANVAVVSGAPLQGQLVARP<br>SSINYMVAPVTGNDVGIRRAEIKQGIREVILCKDQDGKIGLRL<br>KSIDNGIFVQLVQANSPASLVGLRFGDQVLQINGENCAGWS<br>SDKAHKVLKQAFGEKITMTIRDRPFERTITMHKDSTGHVGFIF

	KNGKITSIVKDSSAARNGLLTEHNICEINGQNVIGLKDSQIADI<br>LSTSGTVVTITIMPAFIFEHIIKRMAPSIMKSLMDHTIPEV**
**GFP-APMAP**<br>(Mw: 80 kDa)	MSEADGLRQRRPLRPQVVTDDDGQAPEAKDGSSFSGRVFR<br>VTFLMLAVSLTVPLLGAMMLLESPIDPQPLSFKEPPLLLGVLH<br>PNTKLRQAERLFENQLVGPESIAHIGDVMFTGTADGRVVKLE<br>NGEIETIARFGSGPCKTRDDEPVCGRPLGIRAGPNGTLFVAD<br>AYKGLFEVNPWKREVKLLLSSETPIEGKNMSFVNDLTVTQD<br>GRKIYFTDSSSKWQRRDYLLLVMEGTDDGRLLEYDTVTREV<br>KVLLDQLRFPNGVQLSPAEDFVLVAETTMARIRRVYVSGLMK<br>GGADLFVENMPGFPDNIRPSSSGGYWVGMSTIRPNPGFSM<br>LDFLSERPWIKRMIFKLFSQETVMKFVPRYSLVLELSDSGAF<br>RRSLHDPDGLVATYISEVHEHDGHLYLGSFRSPFLCRLSLQA<br>VGGGGSEQKLISEEDLGGGGSMVSKGEELFTGVVPILVELD<br>GDVNGHKFSVSGEGEGDATYGKLTLKFICTTGKLPVPWPTL<br>VTTLTYGVQCFSRYPDHMKQHDFFKSAMPEGYVQERTIFFK<br>DDGNYKTRAEVKFEGDTLVNRIELKGIDFKEDGNILGHKLEY<br>NYNSHNVYIMADKQKNGIKVNFKIRHNIEDGSVQLADHYQQN<br>TPIGDGPVLLPDNHYLSTQSALSKDPNEKRDHMVLLEFVTAA<br>GITLGMDELYKGGAGGNSRPLEPLEL**
**TSPAN14-GFP**<br>(Mw: 64 kDa)	MHYYRYSNAKVSCWYKYLLFSYNIIFWLAGVVFLGVGLWAW<br>SEKGVLSDLTKVTRMHGIDPVVLVLMVGVVMFTLGFAGCVG<br>ALRENICLLNFFCGTIVLIFFLELAVAVLAFLFQDWVRDRFREF<br>FESNIKSYRDDIDLQNLIDSLQKANQCCGAYGPEDWDLNVYF<br>NCSGASYSREKCGVPFSCCVPDPAQKVVNTQCGYDVRIQL<br>KSKWDESIFTKGCIQALESWLPRNIYIVAGVFIAISLLQIFGIFL<br>ARTLISDIEAVKAGHHFGGGGSEQKLISEEDLGGGGSMVSK<br>GEELFTGVVPILVELDGDVNGHKFSVSGEGEGDATYGKLTLK<br>FICTTGKLPVPWPTLVTTLTYGVQCFSRYPDHMKQHDFFKS<br>AMPEGYVQERTIFFKDDGNYKTRAEVKFEGDTLVNRIELKGI<br>DFKEDGNILGHKLEYNYNSHNVYIMADKQKNGIKVNFKIRHNI<br>EDGSVQLADHYQQNTPIGDGPVLLPDNHYLSTQSALSKDPN<br>EKRDHMVLLEFVTAAGITLGMDELYKGGAGGNSRPLEPLEL*<br>*
**CD63-GFP**<br>(Mw: 58 kDa)	MAVEGGMKCVKFLLYVLLLAFCACAVGLIAVGVGAQLVLSQT<br>IIQGATPGSLLPVVIIAVGVFLFLVAFVGCCGACKENYCLMITF<br>AIFLSLIMLVEVAAAIAGYVFRDKVMSEFNNNFRQQMENYPK

	NNHTASILDRMQADFKCCGAANYTDWEKIPSMSKNRVPDSCCINVTVGCGINFNEKAIHKEGCVEKIGGWLGGGGSGGGGSMVSKGEELFTGVVPILVELDGDVNGHKFSVSGEGEGDATYGKLTLKFICTTGKLPVPWPTLVTTLTYGVQCFSRYPDHMKQHDFFKSAMPEGYVQERTIFFKDDGNYKTRAEVKFEGDTLVNRIELKGIDFKEDGNILGHKLEYNYNSHNVYIMADKQKNGIKVNFKIRHNIEDGSVQLADHYQQNTPIGDGPVLLPDNHYLSTQSALSKDPNEKRDHMVLLEFVTAAGITLGMDELYKGGAGGNSRPLEPLEL**
**CD63TM-GFP**<br>**(CD63-PDGFβ-GFP)**<br>(Mw: 63 kDa)	MAVEGGMKCVKFLLYVLLLAFCACAVGLIAVGVGAQLVLSQTIIQGATPGSLLPVVIIAVGVFLFLVAFVGCCGACKENYCLMITFAIFLSLIMLVEVAAAIAGYVFRDKVMSEFNNNFRQQMENYPKNNHTASILDRMQADFKCCGAANYTDWEKIPSMSKNRVPDSCCINVTVGCGINFNEKAIHKEGCVEKIGGWLRKNVLVVAAAALGIAFVEVLGIVFACCLVKSIRSGYEVMGGGGSEQKLISEEDLVVISAILALVVLTIISLIILIGGGGSMVSKGEELFTGVVPILVELDGDVNGHKFSVSGEGEGDATYGKLTLKFICTTGKLPVPWPTLVTTLTYGVQCFSRYPDHMKQHDFFKSAMPEGYVQERTIFFKDDGNYKTRAEVKFEGDTLVNRIELKGIDFKEDGNILGHKLEYNYNSHNVYIMADKQKNGIKVNFKIRHNIEDGSVQLADHYQQNTPIGDGPVLLPDNHYLSTQSALSKDPNEKRDHMVLLEFVTAAGITLGMDELYKGGAGGNSRPLEPLEL**
**GFP-CD81TM**<br>**(GFP-CD81-PDGFβ)**<br>(Mw: 64 kDa)	MPLLLLLPLLWAGALAMVSKGEELFTGVVPILVELDGDVNGHKFSVSGEGEGDATYGKLTLKFICTTGKLPVPWPTLVTTLTYGVQCFSRYPDHMKQHDFFKSAMPEGYVQERTIFFKDDGNYKTRAEVKFEGDTLVNRIELKGIDFKEDGNILGHKLEYNYNSHNVYIMADKQKNGIKVNFKIRHNIEDGSVQLADHYQQNTPIGDGPVLLPDNHYLSTQSALSKDPNEKRDHMVLLEFVTAAGITLGMDELYKGGAGGNSRPLEPLELGGGGSEQKLISEEDLVVISAILALVVLTIISLIILIGGGGSMGVEGCTKCIKYLLFVFNFVFWLAGGVILGVALWLRHDPQTTNLLYLELGDKPAPNTFYVGIYILIAVGAVMMFVGFLGCYGAIQESQCLLGTFFTCLVILFACEVAAGIWGFVNKDQIAKDVKQFYDQALQQAVVDDDANNAKAVVKTFHETLDCCGSSTLTALTTSVLKNNLCPSGSNIISNLFKEDCHQKIDDLFSGKLYLIGIAAIVVAVIMIFEMILSMVLCCGIRNSSVY**

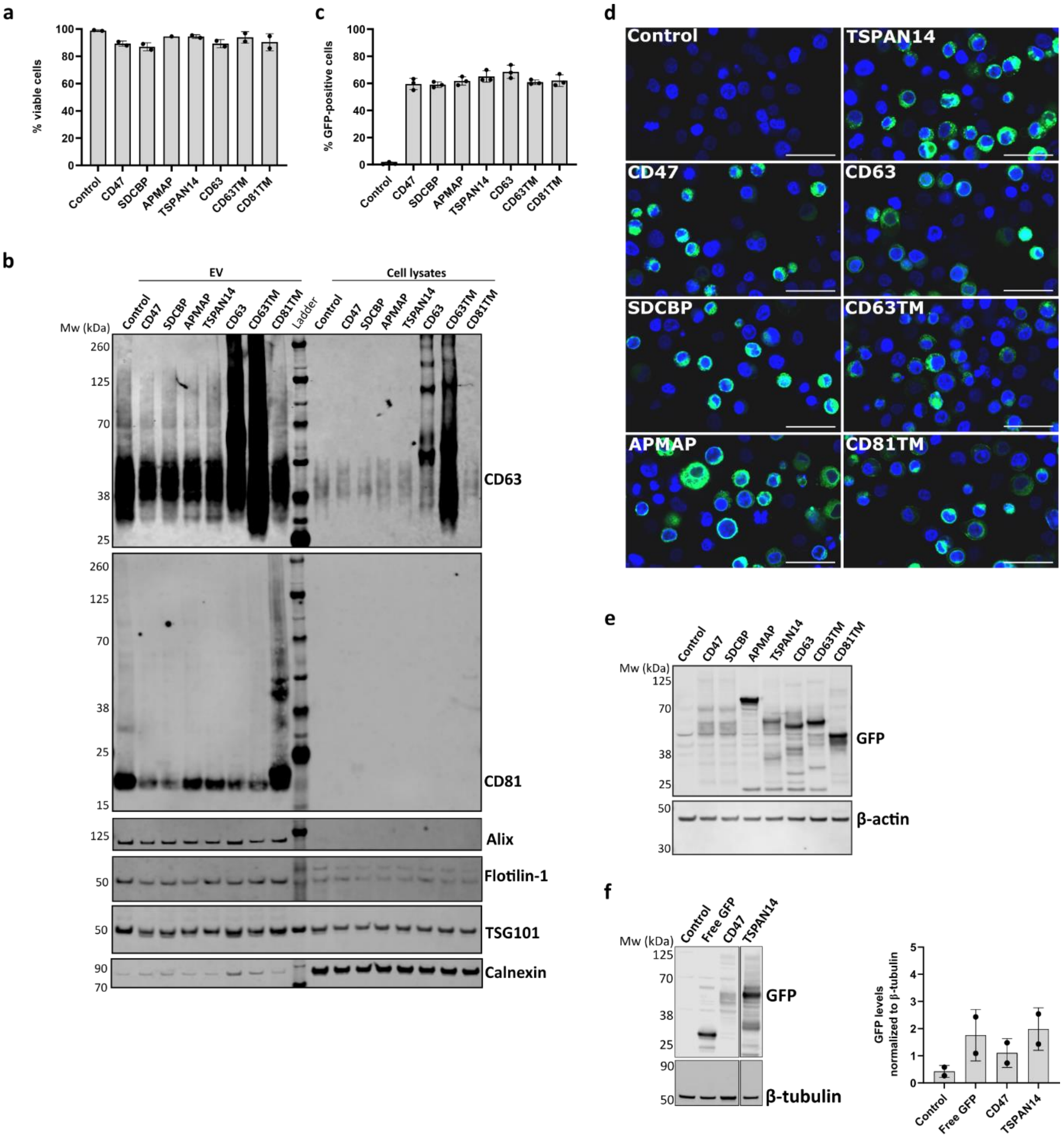

Supplementary Figure 1. Characterization of Expi293F cells engineered with EV-sorting proteins fused to GFP. (a) Viability of Expi293F cells at 48 h post-transfection with the indicated EV-sorting proteins as compared to non-transfected (control). (b) Representative Western blots for the detection of the indicated proteins in Expi293F EVs and corresponding cell lysates. The same amount of protein was loaded for all samples (5 µg/lane). Molecular weight of protein standards is indicated next to the blots. (c) Transfection efficiency of Expi293F cells at 48 h post-transfection with DNA plasmids encoding the indicated EV-sorting proteins, as determined by flow cytometry. (d) Representative micrographs of Expi293F cells transfected with the GFP fusion constructs at 48 h post-transfection, showing the cellular localization of the expressed GFP green fluorescent protein. Scale bar: 50 µm. (e)

Representative Western blot of GFP detection in whole lysates of transfected and control cells loaded at the same protein quantity per lane (25 µg). β-actin was used as loading control. Molecular weight of protein standards is indicated next to the blots. N= 3 biological replicates. (f) Representative Western blots of GFP and β-tubulin detection (left panel) in EVs from Expi293F cells untransfected or engineered with the indicated proteins. The same amount of particles was loaded for all samples ($1.7x10^{10}$ particles/lane). Molecular weight of protein standards is indicated next to the blots. Quantification of GFP levels in EVs across experiments is depicted on right panel, normalized to the levels of β-tubulin. Quantification of the specific bands corresponding to free GFP or the fusion proteins was performed by image analysis of the blots using ImageJ software. N= 2 biological replicates. All graphs represent mean ± standard deviation.

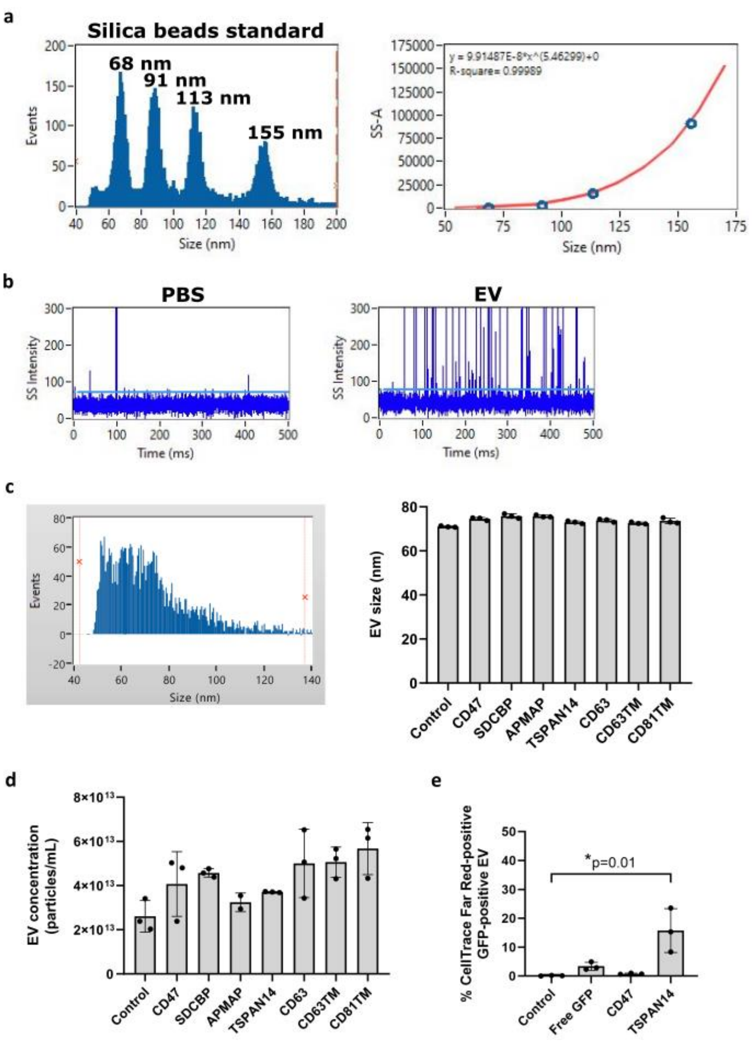

Supplementary Figure 2. Nanoflow cytometry characterization at the single-vesicle level of EVs engineered with GFP-sorting proteins. EVs were isolated by differential ultracentrifugation from Expi293F cells engineered with the indicated EV-sorting proteins fused to GFP, at 48 h after cell transfection, and analysed on NanoAnalyzer N30 nanoflow cytometer (nanoFCM Inc.). (a) Histogram of size distribution of standard silica beads (left panel) used for the calculation of the standard curve for interpolation of the size of the analysed EVs (right panel). (b) Representative histograms of the distribution of side scatter (SSC-A) event-triggered burst for PBS control (left panel) and representative EVs sample (right panel), evidencing the threshold defined (light blue line) to gate the whole EV populations analysed. (c) Representative histogram of size distribution profile (left panel) and average size (right panel) for all the engineered EVs, as determined by Nanoflow cytometry. (d) Average concentration of the engineered EVs analysed, as determined by Nanoflow cytometry. No statistically significant differences were found between conditions. (e) Percentage of GFP^+ EVs secreted by cells overexpressing free, soluble GFP or the GFP fused to the EV-sorting proteins in the whole EV population labelled with the CellTrace™ Far Red dye in the EVs populations secreted by cells overexpressing free, soluble GFP or the GFP fused to the EV-sorting proteins

indicated. P-values are indicated, as determined by Kruskal-Wallis statistical test, followed by Dunn’s correction for multiple comparisons. All graphs represent mean ± standard deviation. N= 3 biological replicates.

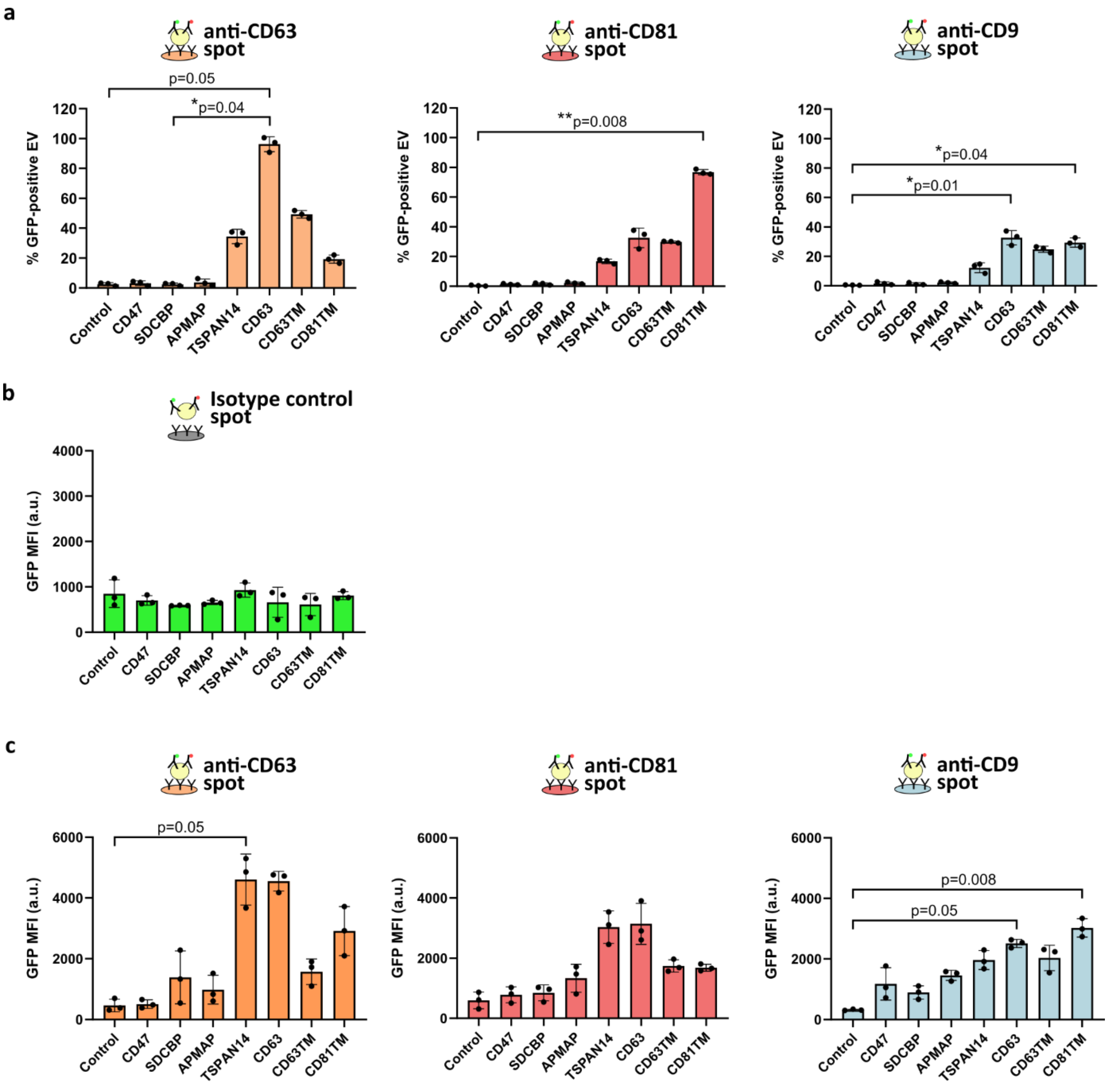

Supplementary Figure 3. Characterization of the tetraspanins content of EVs engineered with sorting proteins fused to GFP, by direct analysis of EV-enriched supernatants using ExoView™. (a) Percentage of GFP^{+} vesicles in EV-enriched supernatant from Expi293F cells engineered with the indicated sorting proteins, as detected in each of the anti-tetraspanins spots present on the ExoView™ chip. Analysis was performed using the supernatant collected after the 20 000 x *g* centrifugation of the Expi293F cell conditioned-media depleted from cell debris, apoptotic bodies and large EVs by differential centrifugation, without further concentration of EVs. P-values are indicated, as determined by Kruskal-Wallis statistical test, followed by Dunn's correction for multiple comparisons. (b) Mean fluorescence intensity of GFP detected in the isotype control spots of the ExoView™ chip, after incubation with the indicated engineered EVs and immunostaining with the anti-tetraspanin and anti-GFP specific antibodies. (c) Mean Fluorescence Intensity (MFI) of GFP in EV-enriched supernatants

for each of the engineered EVs upon capture in the indicated anti-tetraspanin spots of ExoView™ chip. Supernatants were obtained as described above. All graphs represent mean ± standard deviation. N= 3 biological replicates.

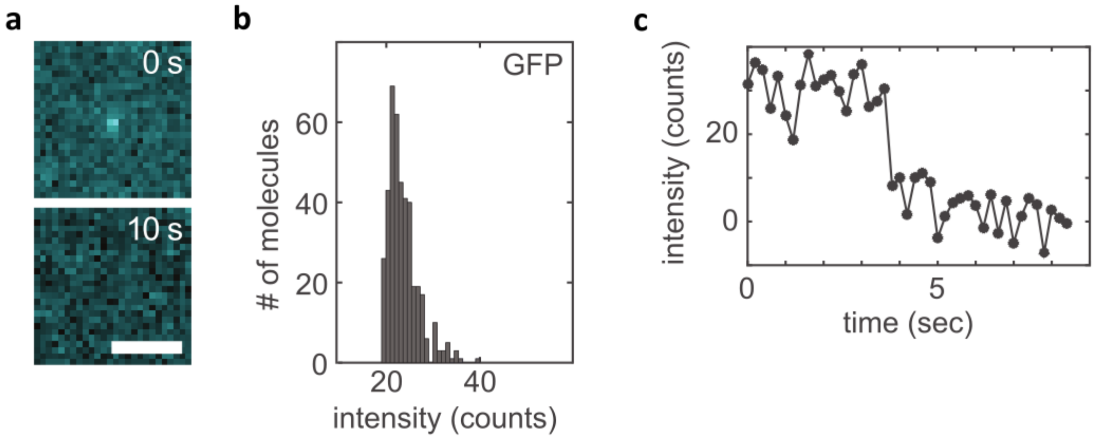

Supplementary Figure 4. Calibration of the Single-Molecule localization microscopy setup for the quantification of GFP copy number in engineered EVs using recombinant GFP. (a) Representative micrographs of GFP photobleaching upon imaging. Scale bar: 30 μm. (b) Fluorescence intensity distribution for different numbers of single GFP molecules. (c) Representative single-step photobleaching profile of GFP over time of imaging.
